# Subjective sleep traits and cognition across mid- to late-adulthood: a cross-sectional study of gene–environment interplay

**DOI:** 10.1093/sleep/zsag119

**Published:** 2026-04-30

**Authors:** Tina T Vo-Eckerle, Nathan A Gillespie, Kaare Christensen, Deborah Finkel, William S Kremen, Marianne Nygaard, Perminder S Sachdev, Anbupalam Thalamuthu, Chandra A Reynolds, for the IGEMS consortium

**Affiliations:** Department of Psychology, University of California, Riverside, Riverside, CA, United States; Department of Psychiatry, University of ia, San Diego, La Jolla, CA, United States; Center for Behavior Genetics of Aging, University of California, San Diego, San Diego, CA, United States; Department of Psychiatry, Virginia Institute for Psychiatric and Behavioral Genetics, Virginia Commonwealth University, Richmond, VA, United States; The Danish Twin Registry, Department of Public Health, University of Southern Denmark, Odense, Denmark; Institute of Gerontology, School of Health and Welfare, Jönköping University, Jönköping, Sweden; Center for Economic and Social Research, University of Southern California, Los Angeles, CA, United States; Department of Psychiatry, University of ia, San Diego, La Jolla, CA, United States; Center for Behavior Genetics of Aging, University of California, San Diego, San Diego, CA, United States; The Danish Twin Registry, Department of Public Health, University of Southern Denmark, Odense, Denmark; Centre for Healthy Brain Ageing (CHeBA), Discipline of Psychiatry and Mental Health, School of Clinical Medicine, UNSW, Sydney, Australia; Centre for Healthy Brain Ageing (CHeBA), Discipline of Psychiatry and Mental Health, School of Clinical Medicine, UNSW, Sydney, Australia; Department of Psychology, University of California, Riverside, Riverside, CA, United States; Institute for Behavioral Genetics, University of Colorado Boulder, Boulder, CO, United States; Department of Psychology and Neuroscience, University of Colorado Boulder, Boulder, CO, United States

**Keywords:** Alzheimer’s disease, polygenic scores, cognitive performance, sleep, gene–environment interplay

## Abstract

Genetic susceptibility to Alzheimer’s disease (AD) may influence the extent to which environmental factors shape cognition, with individuals at higher genetic risk potentially exhibiting greater sensitivity to environmental exposures. Sleep, an important factor for both cognitive function and AD risk, may further moderate genetic influences (A), including both measured (A_P_) and latent (A_L_) components, as well as shared (C) and non-shared environmental (E) contributions to cognition. This study leveraged data from the Interplay of Genes and Environment across Multiple Studies consortium (N = 3894; 1947 complete twin pairs, 842 monozygotic (MZ) pairs and 1105 dizygotic (DZ); Average age = 62.36 years, 38.75% female). Across six cognitive abilities, we examined whether an AD polygenic score (AD-PGS) moderated environmental influences on cognitive performance. We also examined whether sleep moderated genetic and environmental contributions on cognitive performance. Although the AD-PGS accounted for a negligible proportion of genetic variance as a main effect (B’s = –0.004 to 0.02), we observed environment-by-PGS interactions. Increasing genetic risk for AD was associated with lower contributions from environmental experiences unique to each individual, on episodic memory, working memory, and verbal ability (B’s = –0.03 to –0.05). These interaction effects, albeit small, were primarily observed with the AD-PGS including the *APOE* region. Hence, the role of person-specific environments on cognitive functioning was boosted for those at lower genetic risk for AD but reduced at greater genetic risk for AD. Although sleep moderation was minimal, results suggest that poorer sleep influences genetic influences on cognitive functioning.

**Statement of Significance**

This study provides novel insights into how genetic risk for Alzheimer’s disease (AD-PGS), and sleep (duration and disturbances) moderate genetic and environmental contributions to cognitive performance. Although the AD-PGS accounts for minimal genetic variance, significant but small environment-by-PGS interactions emerged. Individuals with higher AD-PGS exhibited heightened responsiveness to nonshared environmental influences on episodic memory, working memory, and verbal ability, primarily driven by the *APOE* ε4 region. Poorer sleep may be associated with subtle variations in genetic contributions to cognition, though moderation effects were minimal. This work offers insight into the genetic mechanisms underlying sleep, AD risk, and cognition, underscoring the need to consider both genetic susceptibility and environmental influences when examining factors that contribute to individual differences in cognition.

## Graphical Abstract

**Graphical Abstract:**
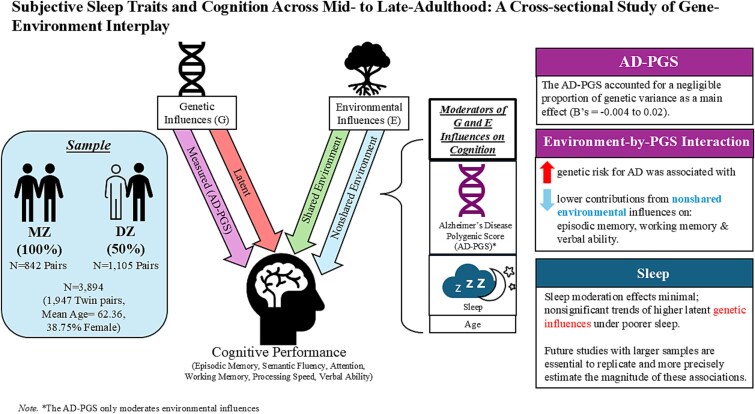

## Introduction

A well-established phenotypic link exists between sleep and cognitive abilities, such that poor sleep—whether due to extreme sleep durations or reduced sleep quality—is associated with lower cognitive functioning across various domains [1–4], as well as with general cognitive decline [5–7]. These associations also extend to an elevated risk of Alzheimer’s disease and related dementias [8–12]. With aging, there is a general decrease in total sleep time, sleep efficiency, and slow-wave sleep, accompanied by an increase in wake after sleep onset, with these disruptions often exacerbated by Alzheimer’s disease (AD) pathology [13, 14]. These sleep changes co-occur alongside normative age-related cognitive declines, such as reasoning, spatial visualization, memory, and processing speed during late life, with varying levels of decline manifesting differentially among individuals [15–18]. However, the etiologic basis of the sleep-cognition association remains unclear. Advances in behavioral genetic methods and the increasing availability of measured genetic data provide new opportunities to examine whether sleep modifies genetic and environmental contributions to cognitive performance and cognitive aging [19]. In particular, sleep may function as a modifiable context that amplifies or attenuates genetic and environmental influences on cognition, consistent with gene–environment interplay frameworks. Yet relatively few studies have tested whether sleep moderates both latent genetic influences and measured genetic susceptibility to AD, and whether genetic susceptibility to AD modifies environmental contributions to cognitive performance. Incorporating polygenic risk for AD (AD polygenic score; AD-PGS) allows us to examine whether AD genetic susceptibility contributes to cognitive performance through (1) measured genetic influences and (2) differential environmental sensitivity, and whether these effects vary across sleep contexts.

Twin analyzes of the genetic architecture of sleep characteristics suggest that sleep quality and sleep duration are moderately heritable, ranging from 30% to 50% and 26% to 70% respectively, although estimates vary depending on the specific sleep measure and method of assessment [20–27]. Relatively few studies have examined sleep as a moderator of the genetic and environmental contributions to cognitive performance. In our prior work [28], sleep duration moderated genetic and environmental contributions to semantic fluency and episodic memory, with genetic influences amplified under shorter sleep duration and attenuated under longer sleep duration. This moderation effect is consistent with the diathesis-stress model which posits that genetic effects on outcomes (e.g. cognition), are heightened under adverse conditions, such as insufficient sleep. Conversely, in more favorable conditions like longer sleep, the impact of genetic influences may diminish. Further examination of gene–environment interplay, particularly in the context of additional sleep features (e.g. sleep disturbances) and measured genetic factors (e.g. AD-PGS), is warranted.

In particular, research underscores the importance of AD-PGS as a broad genetic susceptibility marker for cognitive decline, extending beyond locus-specific effects conferred by the *APOE* ε4 allele, the strongest known genetic risk factor for late-onset AD [29]. Whereas ε4 confers heightened risk, the ε2 allele is generally protective [30]. Higher AD-PGS scores are associated with higher risk of amnestic mild cognitive impairment (MCI) [31], progression from MCI to late-onset AD [32], and cognitive decline [33]. Moreover, higher AD-PGS omitting the *APOE* region has been linked to poorer performance on fluid intelligence tasks and reduced hippocampal and total brain volume [34], suggesting a role for non-*APOE* genetic pathways.

Given links between sleep disturbances and insufficient sleep in the neurobiological pathways implicated in AD (i.e. Aβ accumulation) [35–38] it is important to test whether sleep moderates not only latent genetic influences on cognition but also measured AD-related genetic influences indexed by the AD-PGS. Evidence of moderation would suggest that genetic vulnerability, both in an aggregate, latent form and measured form through the AD-PGS, may be amplified under adverse sleep conditions. This would provide partial insight into the underlying genetic mechanisms contributing to the complex relationship between sleep and cognition. In addition, modeling AD-PGS moderation of shared and nonshared environmental influences enables tests of environment-by-PGS interaction [19]. Specifically, if genetic susceptibility to AD moderates the environmental contributions to cognition, it would suggest that the cognitive effects of environmental exposures, whether protective or detrimental, may differ depending on an individual’s genetic risk for AD. The present study aims to disentangle the ways in which sleep and genetic risk for AD jointly shape cognition, offering a more comprehensive framework for understanding individual differences in cognitive performance.

Despite extensive research on phenotypic links between sleep and cognition, the extent to which sleep and AD genetic susceptibility jointly shape genetic and environmental etiologies underlying cognitive performance remains unclear. We address this gap by leveraging data from the Interplay of Genes and Environment across Multiple Studies (IGEMS) consortium [39], comprising twin pairs in mid- to late-life adulthood. Extending the classical twin model to incorporate measured polygenic influences, we tested whether: (1) AD-PGS moderates environmental contributions to cognitive performance (i.e. environment-by-PGS interaction), and (2) sleep moderates both latent and measured genetic influences, as well as environmental influences, on cognitive outcomes. This approach enables tests of genotype-environment interplay [19, 40, 41] while providing insight into how sleep characteristics modify measured and latent genetic influences and environmental contributions to cognitive outcomes. To further evaluate whether findings were driven specifically by the *APOE* locus or independent of it, we compared AD-PGS models that included versus excluded the *APOE* region. We hypothesized that poor sleep contexts (e.g. increased sleep disturbances or shorter sleep duration) would amplify genetic influences on cognition, consistent with our prior findings, with a particularly strong amplification observed for the genetic effects associated with the AD-PGS. We predicted an environment-by-PGS interaction whereby individuals with higher genetic risk for AD will exhibit greater sensitivity to environmental influences. Lastly, given the distinct biological importance of *APOE,* we anticipated that its inclusion within the AD-PGS would strengthen associations among genetic risk, sleep and cognitive performance.

## Materials and methods

### Sample

The present sample comprised archival data from the IGEMS consortium [39] which encompassed twin pairs spanning mid- to late-life adulthood periods. Specifically, data from seven twin studies were incorporated into the analysis sample and comprised a total of 3894 participants (842 complete pairs of monozygotic (MZ) twins and 1105 complete pairs of dizygotic twins [DZ]). Zygosity was determined through either self-reported questionnaire responses or registry-based information regarding twin and co-twin physical similarities. Zygosity was further validated through DNA analysis and genotyping for select studies [42, 43]. Four MZ twin pairs were discordant on genotype and were excluded from the analyzes. Within the present study, DZ same-sex twins and DZ opposite-sex twins were collapsed into a single DZ group. Furthermore, the analysis sample exclusively included data from twin pairs who both contributed data on the primary moderator variables (i.e. sleep, AD-PGS) and at least one score among the six cognitive tasks. The overall analysis sample ranged from 46 to 92 years old, with an average age of 62.36 (SD = 10.39), and 38.75% female (see Table 1). Detailed descriptions of each study are provided below and available elsewhere [39].

**Table 1:** Demographic characteristics of the sample

Study	N	%Female	# of complete twin pairs		Age	Cognitive tasks
			MZ	DZ	M(SD)	
Total Sample	3894	38.75	842	1105	62.36(10.39)	WL, AN, DF, DB, SYNM, SYMD
SATSA	144	52.78	26	46	74.77(5.82)	WL, DF, DB, SYN, SYMD
OCTO-Twin	58	62.07	22	7	82.34(1.86)	DF, DB, SYN, SYMD
GENDER	366	50	0	183	74.66(2.76)	WL, SYN, SYMD
VETSA	1094	0	314	233	55.95(2.49)	WL, AN, DF, DB, SYN
LSADT	284	70.42	62	80	79.56(3.79)	WL, AN, DF, DB
MADT	1452	47.59	275	451	55.63(5.99)	WL, AN, DF, DB, SYMD
OATS	496	65.12	143	105	71.30(5.48)	WL, DF, DB, SYMD

MZ = monozygotic, DZ = dizygotic, WL = Word List, AN = Animal Naming, DF=Digit Forward, DB=Digit Backward, SYN=Synonyms, SYMD=Symbol Digit, M = mean, SD = standard deviation, SATSA = Swedish Adoption/Twin Study of Aging, OCTO-Twin = Origins of Variance in the Old-Old, GENDER = Aging in Women and Men: A Longitudinal Twin Study of Gender Differences in Health Behavior and Health among Elderly, VETSA = Vietnam Era Twin Study of Aging, LSADT = Longitudinal Study of Aging Danish Twins, MADT = Middle-Aged Danish Twins, OATS=Older Australian Twins Study

### Swedish studies

#### Swedish Adoption Twin Study of Aging

The Swedish Adoption Twin Study of Aging (SATSA), a longitudinal investigation conducted from 1986–2014, compiled data from both reared-together and apart same-sex adult twins over 30 years and across 19 waves of data collection [44]. Participants were recruited from the Swedish Twin Registry. SATSA assessments encompassed questionnaires and in-person assessments (IPT) that assessed cognitive and physical function, along with behavioral evaluations. The analysis sample included data from the 10th in-person assessment (IPT), which included data on participants’ sleep (both duration and disturbances), AD-PGS, and cognitive measures (*n* = 144; 26 complete MZ pairs and 46 complete DZ pairs, 52.78% *F*; see Table 1). The SATSA sample had an age range of 64.21 to 91.37 and an average age of 74.77 (*SD =* 5.82).

#### Origins of Variance in the Old-Old (OCTO-twin)

The Origins of Variance in the Old-Old (OCTO-Twin) longitudinal study, spanning from 1991 to 2002, collected data from same-sex twins aged over 80 years at the baseline assessment [45]. The analysis sample incorporated OCTO-Twin data from the intake wave which included data on participant’s sleep (both duration and disturbances), AD-PGS, and cognitive measures (*n =* 58; 22 complete MZ pairs and 7 DZ pairs, 62.07% *F*; see Table 1). The OCTO-Twin sample had an age range of 79.4 to 86.7 with an average age of 82.34 (*SD =* 1.86).

#### Longitudinal Study of Gender Differences in Health Behavior and Health Among Elderly (GENDER)

The Longitudinal Study of Gender Differences in Health Behavior and Health among Elderly (GENDER) comprised opposite-sex twin pairs, encompassing only DZ twins born between 1906 and 1925 [46]. The analyzes incorporated GENDER data from the first IPT wave which included data on participant’s sleep (only sleep disturbances), AD-PGS, and cognitive measures (*n =* 366; 183 complete DZ pairs, 50% *F*; see Table 1). The GENDER sample had an age range of 69.8 to 80.7 with an average age of 74.66 (*SD =* 2.76).

### Danish studies

#### Middle Age Danish Twins Study

The Middle Age Danish Twins Study (MADT) comprises twin pairs recruited from the Danish Twin Registry [47]. Data for MADT were collected in 1998 or between 2008 and 2011. The analysis sample incorporated MADT data from the intake wave which included data on participants’ sleep (both sleep duration and sleep disturbances), AD-PGS, and cognitive data (*n* = 1452; 275 complete MZ pairs, 451 complete DZ pairs, 47.59% *F*; see Table 1). The MADT sample had an age range of 46.0 to 68.0 years and had an average age of 55.63 (*SD =* 5.99).

#### Longitudinal Study of Aging Danish Twins

The Longitudinal Study of Aging Danish Twins (LSADT) comprises same-sex twin pairs born before 1920, recruited from the Danish Twin Registry [47]. The analysis sample incorporated LSADT data from the intake wave which included data on participants’ sleep (only sleep disturbances), AD-PGS, and cognitive data (*n* = 284; 62 complete MZ pairs, 80 complete DZ pairs, 70.42% *F*; see Table 1). The LSADT sample had an age range of 75.0 to 92.0 years and had an average age of 79.56 (*SD =* 3.79).

### United States study

#### Vietnam Era Twin Study of Aging

The Vietnam Era Twin Study of Aging (VETSA) is a longitudinal study comprising exclusively male twins recruited from the Vietnam Era Twin Registry, who served in the military at sometime between 1965 and 1975 [43]. Nearly 80% of the sample reported no combat exposure. The analysis sample incorporated VETSA data from the first intake wave which included data on participants’ sleep (both sleep duration and sleep disturbances), AD-PGS, and cognitive data (*n* = 1094; 314 complete MZ pairs, 233 complete DZ pairs, 0% *F*; see Table 1). The VETSA sample had an age range of 51.1 to 60.7 years and had an average age of 55.95(*SD* = 2.49).

### Australian Study

#### Older Australian Twins Study

The Older Australian Twins Study (OATS) consisted of Australian twins aged over 65 years and were drawn from the Australian Twin Registry [48]. The analysis sample incorporated OATS data from the first intake wave which included data on participants’ sleep (only sleep disturbances), AD-PGS, and cognitive data (*n* = 496; 143 complete MZ pairs, 105 complete DZ pairs, 65.12% *F*; see Table 1). The OATS sample had an age range of 65.25-90.06 years and had an average age of 71.30 (*SD* = 5.48).

### Measures

#### Cognitive measures

Cognitive tasks evaluating performance in six domains were assessed as outcomes: semantic fluency, episodic memory, attention, working memory, processing speed, and verbal ability (see Table 2). As all cognitive tests involve multiple cognitive processes, assignment to particular cognitive domains varies slightly across studies. Domain assignments were made based on consistency with prior research and harmonization work, and our labeling is consistent with that used in many neuropsychological studies [49, 50]. For example, although Symbol Digit may also involve sustained attention, it was classified under processing speed due to its strong association with timed performance and established use in this domain. Similarly, Digit Span Forward was classified under attention, while Digit Span Backward, which includes mental manipulation, was classified under working memory. To ensure comparability across all studies, cognitive measures were harmonized across the IGEMS samples. Given differences across studies in item number and difficulty, as well as in scoring and administration procedures, it was necessary to place cognitive test scores on a common metric while preserving age-related differences in means and variances. Across the full IGEMS sample, cognitive tests from studies within the same country that had the same testing procedures were pooled. Raw scores on cognitive tasks were then converted to a percent-correct scale to partially account for differences in scoring conventions across studies. Each study sample was divided into four age groups: <50, 50–59.99, 60–69.99, and 70+, with these categories selected to ensure sufficient sample size within each group. The 65–69.99 age group served as the referent for standardizing scores across different tests and studies for each of the cognitive measures. Specifically, within each test-study combination across IGEMS, and after adjusting for the main effect of sex, scores were transformed to a T-score metric which placed all tests on a common scale while preserving variance differences across age groups. Scores within each age group were then winsorized such that values exceeding ±3 standard deviations were capped at ±3 SDs [51]. Brief descriptions of the cognitive tasks are provided below.

**Table 2:** Descriptives of measures by study

Study	Sleep				PGS	Cognitive						
	Sleep duration		Sleep disturbances		AD-PGS	All Cognitive	WL *N* = 3789	AN *N* = 2819	DF *N* = 3516	DB *N* = 3513	SYN *N* = 1641	SYMB *N* = 2345
	*N*	M(SD)	*N*	M(SD)	M(SD)	*Ns*	M(SD)	M(SD)	M(SD)	M(SD)	M(SD)	M(SD)
Overall	2735	7.06 (1.20)	3894	–0.01 (1.06)	–0.0001 (1.04)	1641–3789	51.20 (11.59)	52.74 (10.86)	52.38 (11.29)	51.89 (11.11)	52.95 (8.20)	52.15 (11.11)
SATSA	140	8.79 (1.09)	144	0.13 (1.75)	0.01 (0.98)	141–144	58.08 (13.63)	—	52.76 (12.58)	54.21 (9.62)	53.29 (10.42)	53.04 (11.76)
OCTO-Twin	49	7.31 (1.39)	58	–0.17 (1.14)	–0.02 (0.98)	49–57	—	—	51.32 (11.01)	48.67 (8.51)	46.52 (11.31)	39.53 (11.59)
Gender	—	—	366	–0.22 (1.24)	–0.06 (0.97)	272–359	50.71 (11.83)	—	—	—	50.28 (11.71)	45.96 (10.34)
VETSA	1094	6.64 (1.24)	1094	–0.43 (1.44)	0.01 (1.06)	1086–1094	55.31 (10.48)	53.14 (10.33)	54.94 (11.77)	55.13 (12.62)	54.08 (5.57)	—
LSADT	—	—	284	0.38 (0.49)	0.04 (1.02)	283–284	37.80 (11.83)	42.67 (9.67)	49.48 (10.30)	45.76 (9.31)	—	—
MADT	1452	7.21 (0.95)	1452	0.22 (0.41)	–0.002 (1.04)	1393–1451	51.27 (10.16)	54.42 (10.41)	52.34 (10.84)	51.30 (10.08)	—	55.53 (9.70)
OATS	—	—	496	0.19 (0.82)	0.01 (1.07)	465–491	47.82 (9.52)	—	48.52 (10.13)	49.66 (9.63)	—	46.99 (10.74)

Ns for AD-PGS are equivalent to the Ns for sleep disturbances. Ns for Wordlist (WL) range from 144–1451, Ns for Animal Naming (AN) range from 284–1448, Ns for Digit Forward (DF) range from 57–1451, Ns for Digit Backward (DB) range from 57–1451, Ns for Synonym (SYN) range from 52–1094, Ns for Symbol Digit (SYMD) range from 49–1393. PGS = Polygenic Score, AD-PGS = Alzheimer’s Disease Polygenic Score, SATSA = Swedish Adoption/Twin Study of Aging, OCTO-Twin = Origins of Variance in the Old-Old), GENDER = Aging in Women and Men: A Longitudinal Twin Study of Gender Differences in Health Behavior and Health among Elderly, VETSA = Vietnam Era Twin Study of Aging, LSADT = Longitudinal Study of Aging Danish Twins, MADT = Middle-Aged Danish Twins, OATS=Older Australian Twins Study.

#### Episodic memory (word list)

Episodic memory was assessed using the Word List task from six of the studies (SATSA, GENDER, OATS, VETSA, LSADT, MADT; see Table 2), encompassing the single cognitive task with the largest sample size (N = 3789). Participants were asked to either listen to or read aloud up to 16 related or unrelated words and immediately recall as many words as possible. For the two Swedish samples (SATSA and GENDER), episodic memory was assessed through the Consortium to Establish a Registry for Alzheimer’s Disease instrument (CERAD) [52]. For the two Danish samples (LSADT, MADT) and the Australian sample (OATS), episodic memory was assessed through the Rey Auditory Verbal Learning Test. For the United States study (VETSA), episodic memory was assessed through the California Verbal Learning Test-Version II [53]. The average T-score for the Word List task within the analysis sample was 51.20 (*SD =* 11.59).

#### Semantic fluency (animal naming)

Semantic fluency was assessed using the Animal Naming task from four of the studies (VETSA, LSADT, MADT, OATS; see Table 2). Participants were asked to name as many animals as possible within one minute without any duplicates. The average T-score for the Animal Naming task within the analysis sample was 52.74 (*SD =* 10.86).

#### Attention (digits forward)

Attention was assessed using the Digits Forward task from six of the studies (SATSA, OCTO-Twin, OATS, VETSA, LSADT, MADT; see Table 2). Participants were asked to verbally repeat increasing strings of digits in the same sequence in which they were presented. For the Swedish samples (SATSA, OCTO-Twin), the digits forward task was administered through a modified Wechsler Adult Intelligence Scale (WAIS) with a maximum possible score of 9. For the United States sample (VETSA), the digits forward task was administered through the third edition of the Wechsler Memory Scale (WMS-III) which had a maximum score of 11. For the two Danish samples (LSADT and MADT), the digits forward task was administered through the WAIS which had a maximum score of 14. Lastly, for the Australian sample (OATS), the digits forward task was administered through the third edition of the Wechsler Adult Intelligence Scale (WAIS-III) which had a maximum score of 9. The average T-score for the Digits Forward task in the analysis sample was 52.38 (*SD* = 11.29).

#### Working memory (digits backward)

Working memory was assessed through the Digits Backward task from six of the studies (SATSA, OCTO-Twin, OATS, VETSA, LSADT, MADT). Participants were asked to verbally repeat digits in the reverse sequence in which they were presented. Tests used were the same as for digits forward. For the Swedish Samples (SATSA, OCTO-Twin), the digits backward task maximum possible score was 8. For the two Danish samples (LSADT, MADT), the maximum score was 14. For the United States study (VETSA), the maximum score was 10. Lastly, in the Australian sample (OATS) the maximum score was 8. The average T-score for the Digits Backward task in the analysis sample was 51.89 (*SD* = 11.11).

#### Verbal ability (synonyms)

Verbal ability was assessed in four of the studies (SATSA, OCTO-Twin, GENDER, VETSA), encompassing the single cognitive task with the lowest sample size (*N* = 1641). Participants were given a target word and were tasked with providing a selection of a corresponding synonym within a list of provided options. The Swedish samples (SATSA, OCTO-Twin, and Gender) administered the Synonyms task through the Swedish WAIS [54] which had a maximum possible score of 30. The United States sample (VETSA) administered the Synonyms task through the Armed Forces Qualification Test subscale which had a maximum score of 25. The average T-score for the Synonyms task was 52.95 (*SD =* 8.20).

#### Processing speed (symbol digit)

Processing speed and accuracy were assessed through the Symbol Digit task from four of the studies within our analysis sample (SATSA, OCTO-Twin, GENDER, MADT). Within the Swedish (SATSA, OCTO-Twin, GENDER) and Danish sample (MADT), participants were tasked to verbally state a single digit, ranging from one to nine, that corresponded with a specifically assigned symbol. Participants were instructed to respond as quickly and accurately as possible within a 45-second time limit per block, across two blocks. The average T-score for the Symbol Digit task was 52.15 (*SD* = 11.11).

### Sleep measures

#### Sleep duration

Sleep duration within IGEMS was evaluated through self-reported measures, either by indicating the length of sleep on an average night or by reporting the timing of sleep onset and awakening. To ensure consistency across the diverse range of questions related to sleep duration across the various studies within IGEMS, a harmonization procedure was utilized, resulting in a single measure of sleep duration for each individual [28]. In instances where sleep duration was assessed through multiple items, such as across different seasons or weekdays versus weekends, an average was computed to establish a single sleep duration score. For SATSA participants, sleep duration was computed based on average bedtime and waketime. For VETSA participants, sleep duration information was obtained from a single item. Based on guidelines outlined by the National Sleep Foundation, extremely short and long sleep outliers were winsorized at three standard deviations of age-specific sleep norms.

#### Sleep disturbances

Sleep disturbances were assessed through participant self-reports, utilizing a range of questions aimed at gauging sleep quality across IGEMS. Given the variety and quantity of sleep disturbance items across the IGEMS studies (see Supplemental Tables S1–S2), a harmonized measure of sleep disturbances was created to result in a single, consolidated metric of sleep disturbance. The harmonization process involved consolidating self-report items related to sleeping problems, sleep apnea, insomnia, nightmares, sleep medication usage, restlessness, snoring, and problems related to sleep maintenance into a binary classification of either no endorsement or some endorsement. These items were categorized based on clinical information from the 2020 ICD-10 and also aligned with similar items and subcategories within the Pittsburgh Sleep Quality Index [55]. Confirmatory factor analysis (CFA) in MPLUS version 8.6 was used to generate factor scores based on all available items for each individual across the full IGEMS sample (N = 12 006; see Supplemental Table S2), following a similar approach to previous IGEMS work [56]. CFA was selected to formally model the shared variance across heterogeneous sleep items, test a theoretically based structure of sleep disturbances across multiple studies, account for measurement error, and ensure that the latent construct is consistently captured despite item heterogeneity. As the factors resulting from the two-factor model were highly correlated (r = 0.94), we proceeded with a one-factor model. Factor scores were then rank normalized to reduce nonlinearity and skew such that skewness prior to rank normalization was 0.71 and skewness post-rank normalization was 0.06.

### AD polygenic score

Subsets of individuals within the larger IGEMS consortium had available genome-wide genotype data. The AD-PGS was generated using summary statistics from a large-scale genome-wide association study (GWAS) of clinical AD cases [57]. The procedure for constructing the PGS began by quantifying the number of effect alleles (ranging from 0 to 2) at single nucleotide polymorphisms (SNPs) across the genome and weighting them by the SNP’s effect size from the GWAS summary statistics [57, 58]. To account for linkage disequilibrium (i.e. non-random association of alleles at two or more genetic loci), the SBayesR shrinkage approach was applied, yielding modified effect size weights [58]. The resulting PGS was calculated as the sum of the weighted alleles, representing the aggregated genetic contribution to AD risk.

We computed AD-PGS both including and excluding the SNPs in the *APOE* region which are in strong linkage disequilibrium (CHR19: 44400000-46 500 000, build 37/hg19) [59]. In cases where only one twin from a MZ pair was genotyped, the PGS of the co-twin was based on the genotyped twin’s PGS. The AD-PGS scores were adjusted within country for the first 10 ancestry principal components using a regression procedure to obtain residual scores that were then standardized (M = 0, SD = 1).

### Statistical analyzes

Phenotypic and genetic twin models were fitted in OpenMx version 2.21.8 in R version 4.3.2 [60, 61]. Measures of association including phenotypic and twin correlations were estimated by zygosity group (MZ and DZ; see Table 3). To firstly establish the relationship between sleep, cognition, and AD-PGS, phenotypic correlations were estimated within the full sample (see Table 3). Next, we examined twin pair correlations, comparing the similarity of MZ twins, who share 100% of their genetic information, and DZ twins, who share approximately 50% of their segregating genes, to provide preliminary evidence regarding the relative contributions of genetic and environmental factors to sleep and cognitive traits. To partition the genetic and environmental influences, univariate twin models were applied to the data, which leverage the distinctive features of the classical twin design to estimate various baseline sources of variation stemming from both genetic and environmental influences. These latent sources include additive genetic influences (A_L_) which make twins more alike (correlated at 1.0 for MZ twins and 0.5 for DZ twins), shared environmental influences (C) such as shared family environments which make twins, regardless of zygosity, more similar (correlated at 1.0 for both), and non-shared environmental influences (E), encompassing individual-specific factors that make twins more dissimilar and measurement error (uncorrelated for both twin types).

**Table 3:** Phenotypic, twin correlations, and univariate standardized variance estimates

		Phenotypic correlations			Twin correlations		Univariate standardized variance estimates		
Trait		Sleep Duration	Sleep Disturbances	AD-PGS	MZ	DZ	SA	SC	SE
							[95% CIs]	[95% CIs]	[95% CIs]
Word List	*r*	–0.04^*^	–0.07^**^	**–**0.03	0.40^**^	0.28^**^	0.23	0.17	0.6
	*N*	2677	3789	3789	800	1081	[0.07, 0.38]	[0.05, 0.29]	[0.55, 0.66]
Animal Naming	*r*	**–**0.009	–0.06^**^	**–**0.03	0.46^**^	0.28^**^	0.41	0.07	0.52
	*N*	2535	2819	2819	644	7620	[0.25, 0.58]	[**–**0.07, 0.20]	[0.47, 0.58]
Digit Forward	*r*	**–**0.02	–0.07^**^	0.004	0.48^**^	0.28^**^	0.38	0.09	0.53
	*N*	2728	3516	3516	835	919	[0.22, 0.53]	[**–**0.03, 0.22]	[0.48, 0.58]
Digit Backward	*r*	–0.04^*^	–0.07^**^	0.02	0.45^**^	0.24^**^	0.4	0.05	0.56
	*N*	2727	3513	3513	832	920	[0.24, 0.55]	[**–**0.08, 0.17]	[0.51, 0.61]
Synonyms	*r*	–0.07^*^	–0.04^*^	**–**0.02	0.68^**^	0.37^**^	0.62	0.06	0.32
	*N*	1277	1641	1641	358	454	[0.45, 0.79]	[**–**0.09, 0.20]	[0.27, 0.39]
Symbol Digit	*r*	–0.06^*^	**–**0.02	**–**0.02	0.72^**^	0.50^**^	0.43	0.29	0.28
	*N*	1573	2345	2345	428	692	[0.30, 0.56]	[0.17, 0.40]	[0.25, 0.33]
Sleep Duration	*r*		–0.14^**^	**–**0.01	0.37^**^	0.29^**^	0.13	0.24	0.63
	*N*		2735	2735	629	733	[**–**0.05, 0.31]	[0.09, 0.38]	[0.57,0.70]
Sleep Disturbance	*r*			**–**0.01	0.23^**^	0.19^**^	0.05	0.17	0.78
	*N*			3894	842	1105	[**–**0.12, 0.22]	[0.04, 0.30]	[0.72, 0.84]

^*^ *p* < .05, ^**^  *p* < .01, twin correlations and univariate standardized variance estimates were adjusted for age and sex. AD-PGS = Alzheimer’s Disease Polygenic Score, MZ = monozygotic, DZ = dizygotic, SA = heritability, SC = common environmentality, SE = nonshared-environmentality. Boldface values indicate statistically significant associations (p < .05), consistent with the asterisk notation used in the table.

Univariate ACE twin models also provided a baseline decomposition of the underlying genetic and environmental contributions before the inclusion of moderators (see Table 3 and Supplemental Table S3). Of note, negative confidence intervals are possible under univariate ACE estimates. Further, we assessed whether assumptions for biometrical modeling were met. This involved conducting separate equality tests for means and variances across the sleep and cognitive measures. Specifically, equality tests for means and variances were conducted in successively nested models: (1) means and variances were unconstrained across twin order or zygosity, (2) means were constrained across twins within zygosity groups, (3) means and variances were constrained across twins within zygosity group, and (4) means and variances were constrained across both twin and zygosity group. The Log-likelihood ratio test (LRT) model comparisons for these tests yielded non-significant results (all *p*-values ≥ .11).

Building upon these univariate models, bivariate twin models were applied to sleep and cognition to decompose the genetic and environmental variance unique to sleep (i.e. a1, c1, e1) and unique to cognition (i.e. a2, c2, e2), as well as the sources of covariation between the two phenotypes (i.e. a21, c21, e21; see Supplemental Table S4) [7, 62–63]. Establishing this baseline relationship is essential for three reasons. First, it allows us to determine whether sleep and cognition share common genetic and/or environmental etiologies, information that is crucial for interpreting the potential impact of sleep as a moderator. Second, evidence of a significant main effect of sleep on cognition would justify the inclusion of sleep as a moderator in subsequent extended moderation models [19]. Specifically, a main effect of sleep is determined if the removal of the sources of covariation (a21, c21, and e21) results in a significant reduction in model fit. Note however, a main effect of sleep is not explicitly necessary to test for moderation effects [64]. Finally, by confirming the baseline genetic/environmental overlap between these traits, we can test whether the bivariate relationship is altered by additional moderators.

**Figure 1:**
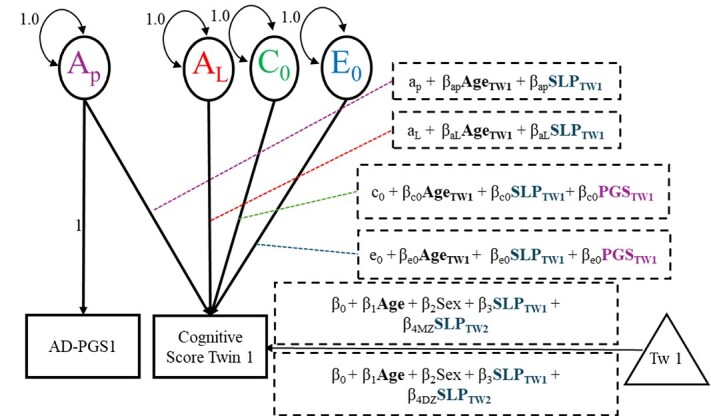
Biometrical ACE moderation model of AD-PGS, age, and sleep effects on genetic and environmental influences on cognition. *Note.* Path diagram showing how AD-PGS (Alzheimer’s disease polygenic score), age, and sleep moderate genetic and environmental influences on cognition, adapted from Bruins et al. (2022). Model shown for one twin from a twin pair. Circles represent latent variables, squares indicate observed variables, and triangle represents the mean cognitive score/trait. Ap = additive genetic influence specific to AD-PGS & covarying with cognition; AL, C0, & E0 = additive genetic, shared & non-shared environmental influences unique to cognition; SLP=sleep covariate; PGS=polygenic score. Equations in dashed boxes show moderating effects of age and sleep on all genetic & environmental sources of variation; the moderating effects of AD-PGS are only modeled on C0 and E0.

To test our core hypotheses concerning whether measured genetic risk for AD, sleep, and age moderate individual differences in cognition, we applied an extension of the model proposed by Bruins et al. [19]. This model was developed to evaluate not only the main effects of PGSs on a trait, but also whether PGSs moderate latent sources of environmental influences (C_0_ and E_0_) underlying complex traits. Our extension further allows age and sleep to moderate all three latent sources of unique variation (A_L_, C_0_, E_0_), as well as the main effects of the measured AD-PGS on cognition (A_p_; see Figure 1).^1^

Specifically, the model partitions residual variance in cognition, after adjustment for covariates, into four components: variance attributable to the AD-PGS (A_p_), latent additive genetic influences not captured by the AD-PGS (A_L_), shared environmental influences (C_0_), and nonshared environmental influences (E_0_) (see Figure 1). We examined whether C and E influences were moderated by the AD-PGS, sleep duration or sleep disturbances, and age. By design, the AD-PGS was specified to interact only with the environmental influences underpinning cognitive performance (C_0_ and E_0_; see Figure 1), reflecting the fact that the measured genetic influence (i.e. the AD-PGS; A_p_) cannot moderate the latent genetic influence (A_L_). In contrast, age and sleep are allowed to moderate the main effect of the AD-PGS (A_p_), as well as all latent contributions (A_L_, C_0_, E_0_) to cognitive performance (see Figure 1).

In all models, the effects of age, sex, sleep, and country-level effects were also included as definition variables in OpenMx to adjust the cognitive performance latent mean as covariates. Moreover, co-twin sleep was also included as a definition variable and contributed to the mean level prediction by zygosity to adjust for differential MZ and DZ twin resemblance on the sleep moderator [65]. Covariates of age, sex, and country were constrained across twins and zygosity types. Country-level effects were controlled based on a series of dummy codes assigned to studies representing Sweden, the United States, and Australia. Within these dummy codes, studies from Denmark were the reference group as they represented the largest sample and were the most represented across the various cognitive tasks. However, for one cognitive measure (i.e. Synonyms), the United States sample was the referent group as only the Swedish studies and the United States study contributed samples. The residual variance of cognition, following the regression of covariates on the mean-level of cognition, was then partitioned into A_P_, A_L_, C, and E components of variance, allowing for these residual components to vary as a function of the moderators (see Figure 1). Moderation effects therefore reflect changes in the magnitude of genetic, both measured and latent, contributions (for sleep and age moderation) and environmental contributions (for AD-PGS, sleep, and age moderation) to cognitive performance across levels of the moderators.

Significant moderators were determined based on estimating 95% confidence intervals and model fit (see Supplemental Tables S5 and S6). Of note, Wald *p*-values and false discovery rate (FDR)-corrected *p*-values are also provided. However, profile likelihood confidence intervals are generally preferred and more accurate in structural equation modeling analyzes because they account for the nonlinear nature of the parameter space and likelihood surface, which may not be symmetric or normally distributed. Accordingly, we rely primarily on profile likelihood confidence intervals and LRTs when determining significance. Of note, data preprocessing included the standardization of all moderators and outcomes (i.e. z-scored, M = 0, SD = 1). Model fit was evaluated using both the LRT and Akaike’s Information Criterion (AIC) [66]. The LRT assesses the goodness-of-fit between a fuller model and a nested sub-model, with differences in model fits distributed as a chi-square (**χ**^2^) under the null hypothesis. AIC was calculated as -2(log-likelihood) +2 K, where K represents the number of parameters within the model. AIC comparisons for model fit may be made in both nested and unnested model comparisons and AIC provides a penalty-adjusted fit function for model complexity. Models with lower AIC values were considered better fitting models.

## Results

### Descriptive statistics

The average sleep duration for the total analysis sample was 7.06 hours (*SD* = 1.20, *N* = 2735; see Table 2), varying from 6.64 hours (SD = 1.24) in VETSA to 8.79 hours (SD = 1.09) in SATSA, with the largest variation in sleep duration observed for OCTO-Twin (SD = 1.39). The average sleep disturbance factor score was –0.01 (SD = 1.06), varying from -0.43 (SD = 1.44) in VETSA to 0.38 (SD = 0.49) in LSADT, with the largest variation in sleep disturbance factor scores observed for SATSA (SD = 1.75). Overall, sleep duration within this sample is normative such that the majority of individuals slept within the recommended 7 to 9 hours for this mid-to-late-life age group based on National Sleep Foundation norms. Sleep disturbances were normally distributed, with a skewness of 0.06 post-rank normalization. Additional descriptive statistics on the sleep measures, the AD-PGS, and the cognitive measures are depicted in Table 2.

### Correlations

Phenotypic correlations between the sleep measures and cognitive performance are depicted in Table 3. Both sleep duration and sleep disturbances tended to have small negative correlations with cognitive performance. For sleep duration associations with cognition, statistically significant but nominal associations were observed for Digits Backward (*r* = –0.04, *p <* .*001*), Word List (*r =* –0.04, *p* < .001), Symbol Digit (*r* = –0.06, *p <* .001) and Synonyms (*r =* –0.07, *p* < .001), with nonsignificant associations observed for Animal Naming (*r* = –0.01, *p* > .05) and Digit Forward (*r* = –0.02, *p* > .05). Sleep disturbance exhibited negative associations across most cognitive tasks (median *r* = –0.07 sleep disturbances vs median *r* = –0.04 sleep duration). Moreover, sleep disturbances were negatively associated with sleep duration (*r =* –0.14, *p <* .001). Nonsignificant associations were observed between the sleep measures and the AD-PGS. Similarly, associations between the AD-PGS and cognition were nonsignificant but generally in the expected direction of increased genetic risk for AD and poorer cognitive performance.

MZ and DZ twin pair correlations for each trait, adjusted for age and sex, are depicted in Table 3. For sleep duration, the MZ correlation was *r* = 0.37, *p* < .0001 and the DZ correlation was *r* = 0.29, *p* < .0001, with similar albeit weaker patterns for sleep disturbances (*r*_MZ_ = 0.23, *p* < .0001, *r*_DZ_ = 0.19, *p* < .0001). Twin correlations for MZ and DZ pairs for each cognitive task ranged from 0.40 to 0.72 for MZ pairs and 0.24 to 0.50 for DZ pairs. These patterns of twin correlations suggest that genetic influences may contribute to variability in the sleep measures as well as cognition (*r*_MZ_ > *r*_DZ_), while still indicating substantial environmental influences.

### Genetic and environmental influences

Standardized univariate ACE estimates, adjusted for age and sex, for the sleep measures and the cognitive measures are provided in Table 3 and Supplemental Table S3. These standardized ACE estimates describe the estimates of heritability (SA), common environmentality (SC), and nonshared environmentality (SE) of the sleep and cognitive measures. For the sleep measures, the heritability estimates were small. Additive genetic effects accounted for 13% percent of the variance in sleep duration and roughly 5% of the variance in sleep disturbances. Of note, the 95% CIs overlapped with zero. In contrast, the point estimates for the shared environmental influences were significant. The heritability estimates for the cognitive measures were moderate, with the highest estimates observed for Synonyms (62%) and the lowest observed for Word List (23%).

Bivariate ACE models between sleep duration and cognitive tasks showed no significant main effects of sleep duration as dropping the paths specific to genetic (a21), shared environmental (c21), and non-shared environmental (e21) influences of sleep on cognition resulted in a negligible change in model fit (all *p’s* > 0.05; see Supplemental Table S4). Bivariate ACE models between sleep disturbances and cognitive performance, do suggest a significant main effect of sleep disturbances for Wordlist (**χ**^2^(3) = 15.49, *p* = .001), Digits Forward (**χ**^2^(3) = 9.17, *p* = .03), and Digits Backward (**χ**^2^(3) = 14.33, *p* = .002), but not for Animal Naming, Symbol Digit, or Synonyms (all *p’s* > 0.05). Importantly, although main effects of sleep on cognition were minimal or nonsignificant, this does not preclude meaningful interactions; moderation can occur even in the absence of direct associations, reflecting how sleep may influence the magnitude of genetic and environmental contributions to cognitive performance [67].

#### Moderation models

Informed by the bivariate models, we present the parameter estimates from the better-fitting AE models in the main text (see Table 4). Parameter estimates from the ACE models are presented in the supplement as well as model fit comparisons (see Supplemental Tables S6–S13).

**Table 4:** Parameter estimates and 95% Confidence Intervals for AE models: AD-PGS, age, and sleep disturbances as moderators

Cognitive Task	Est.	95% CI FDR *p*-value	Est.	95% CI FDR *p*-value	Est.	95% CI FDR *p*-value
***Word List***	A_p_		A_L_		E_0_	
	**–0.04**	**[–0.07, –0.01]**	**0.48**	**[0.42, 0.53]**	**0.77**	**[0.74, 0.80]**
AD-PGS					B_Ap,E_	
	—	—	—	**—**	**–0.03**	**[–0.05, –0.003]**
						**0.05**
Age	B_Age,Ap_		B_Age,AL_		BA_ge,E_	
	**–0.04**	**[–0.07, –0.01]**	**0.09**	**[0.04, 0.14]**	**–**0.01	[**–**0.04, 0.02]
		**0.05**		**0.01**		0.43
Sleep Dist.	B_SD,Ap_		B_SD,AL_		B_SD,E_	
	**–**0.004	[**–**0.03, 0.03]	0.04	[**–**0.03, 0.11]	**–**0.03	[**–**0.07, 0.01]
		0.78		0.32		0.32
***Animal Naming***	A_p_		A_L_		E_0_	
	**–**0.03	[**–**0.06, 0.01]	**0.59**	**[0.54, 0.64]**	**0.73**	**[0.70, 0.77]**
AD-PGS					B_Ap,E_	
	—	—	—	—	**–**0.01	[**–**0.04, 0.01]
						0.60
Age	B_Age,Ap_		B_Age,AL_		BA_ge,E_	
	**–**0.01	[**–**0.05, 0.03]	0.01	[**–**0.04, 0.06]	**–0.04**	**[–0.07, –0.004]**
		0.77		0.83		0.16
Sleep Dist.	B_SD,Ap_		B_SD,AL_		B_SD,E_	
	0.03	[**–**0.01, 0.06]	0.01	[**–**0.06, 0.07]	0.03	[**–**0.02, 0.07]
		0.49		0.84		0.60
***Digit Forward***	A_p_		A_L_		E_0_	
	0.002	[**–**0.03, 0.04]	**0.67**	**[0.62, 0.71]**	**0.72**	**[0.69, 0.75]**
AD-PGS					B_Ap,E_	
	—	—	—	—	**–**0.01	[**–**0.03, 0.02]
						0.56
Age	B_Age,Ap_		B_Age,AL_		BA_ge,E_	
	**–**0.01	[**–**0.05, 0.03]	**–**0.01	[**–**0.06, 0.03]	**–**0.01	[**–**0.04, 0.02]
		0.56		0.56		0.56
Sleep Dist.	B_SD,Ap_		B_SD,AL_		B_SD,E_	
	**–**0.01	[**–**0.05, 0.02]	0.05	[**–**0.01, 0.1]	**–**0.03	[**–**0.07, 0.01]
		0.56		0.54		0.54
***Digit Backward***	A_p_		A_L_		E_0_	
	0.02	[**–**0.02, 0.05]	**0.61**	**[0.57, 0.66]**	**0.74**	**[0.71, 0.77]**
AD-PGS					B_Ap,E_	
	—	—	—	**—**	**–0.03**	**[–0.05, –0.001]**
						0.13
Age	B_Age,Ap_		B_Age,AL_		BA_ge,E_	
	-0.004	[**–**0.04, 0.03]	**–0.06**	**[–0.11, –0.01]**	**–**0.03	[**–**0.06, 0.005]
		0.80		0.09		0.20
Sleep Dist.	B_SD,Ap_		B_SD,AL_		B_SD,E_	
	0.02	[**–**0.01, 0.05]	0.04	[**–**0.04, 0.11]	**–**0.04	[**–**0.08, 0.01]
		0.28		0.39		0.26
***Synonyms***	A_p_		A_L_		E_0_	
	**–**0.02	[**–**0.08, 0.03]	**0.68**	**[0.63, 0.74]**	**0.63**	**[0.59, 0.67]**
AD-PGS					B_Ap,E_	
	—	—	—	**—**	**–0.05**	**[–0.08, –0.02]**
						**0.01**
Age	B_Age,Ap_		B_Age,AL_		BA_ge,E_	
	**–**0.05	[**–**0.11, 0.01]	**0.34**	**[0.27, 0.41]**	**0.10**	**[0.05, 0.15]**
		0.17		**0.00**		**0.00**
Sleep Dist.	B_SD,Ap_		B_SD,AL_		B_SD,E_	
	**–**0.02	[**–**0.05, 0.02]	0.01	[**–**0.04, 0.07]	0.03	[**–**0.01, 0.07]
		0.41		0.62		0.28
***Symbol Digit***	A_p_		A_L_		E_0_	
	**–**0.03	[**–**0.07, 0.01]	**0.70**	**[0.66, 0.74]**	**0.52**	**[0.49, 0.56]**
AD-PGS					B_Ap,E_	
	—	—	—	—	0.01	[**–**0.02, 0.04]
						0.83
Age	B_Age,Ap_		B_Age,AL_		BA_ge,E_	
	0.003	[**–**0.04, 0.04]	**0.08**	**[0.04, 0.12]**	0.02	[**–**0.01, 0.05]
		0.88		**0.00**		0.51
Sleep Dist.	B_SD,Ap_		B_SD,AL_		B_SD,E_	
	0.01	[**–**0.02, 0.05]	**–**0.03	[**–**0.07, 0.02]	0.02	[**–**0.02, 0.07]
		0.66		0.51		0.51

Ap = genetic influences attributable to the AD-PGS, AL = additive genetic influences, E = non-shared environmental influences, Est. = Estimate. Moderation parameter terms include the AD-PGS, age, and sleep disturbances. Bolded parameters indicate *p* < .05.

#### Sleep duration

**Figure 2:**
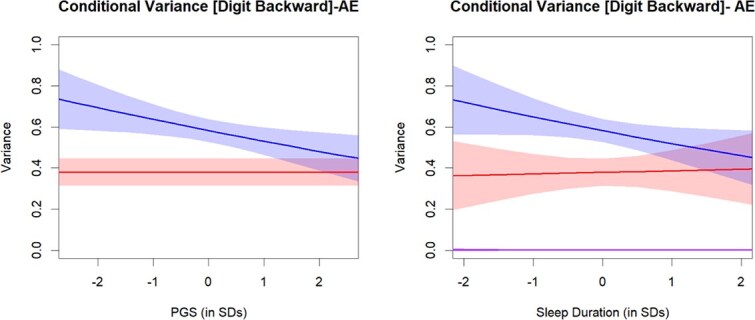
Genetic and environmental variance of digit backward, across varying levels of the AD-PGS and varying levels of sleep duration. *Note.* Red line = additive genetic variance AL, purple line = proportion of measured additive genetic variance AP attributable to the AD polygenic score (AD-PGS), blue line = nonshared environmental variance E. Conditional plots were generated from the fitted AE model by computing model-implied latent additive genetic (AL), measured additive genetic (AP), and unique environmental (E) contributions across values of the focal moderator. Shading depicts 95% confidence intervals. For each moderator value (e.g. AD polygenic score or sleep, expressed in SD units), other moderators were fixed at their mean (0), illustrating how model-implied effects change across the range of the moderator. Panels depict results from the AE model.

Among the three moderators of the genetic and environmental influences on Digits Backward (sleep duration, AD-PGS, and age), sleep duration significantly moderated the E parameter (AE Model; B = -0.04, 95% CI = -0.09, -0.001, Wald *p* = 0.049, FDR-corrected *p* = 0.11; see Supplemental Table S7 and Figure 2). This negative coefficient suggests that the influence of unique environmental factors (i.e. those environmental experiences unique to each individual, not shared between twins) in working memory decreases with longer sleep. This attenuation may reflect a reduced variability in environmental impacts on cognition when sleep duration is extended. The AD-PGS (A_p_) main effect on Digits Backward was not significant (B = 0.02, 95% CI = –0.02, 0.06) suggesting no direct contribution of the AD-PGS to Digits Backward. Rather, it is only through the PGS’ interplay and interaction with the nonshared environment that the AD-PGS plays a role (AE Model; B = –0.04, 95% CI = –0.06, –0.01, Wald *p* = 0.02, FDR-corrected *p =* 0.11; see Supplemental Table S7 and Figure 2), suggesting an attenuation of the contributions from the unique environmental influences on Digits Backward with higher genetic risk for AD. Similar patterns regarding the AD-PGS interaction with the nonshared environment were observed for Word List (AE Model; B = -0.03, 95% CI = –0.06, –0.01, Wald *p* = 0.01, FDR-corrected *p* = 0.04) and for Synonyms (AE Model; B = –0.07, 95% CI = –0.11, –0.02, Wald *p* = 0.002, FDR-corrected *p* = 0.01). Sleep duration did not significantly moderate A_p_, the portion of the genetic variance that was indexed by the AD-PGS, nor did it moderate the portion of the genetic variance of the latent genetic influence (A_L_) on differences in cognitive performance.

Sleep duration moderation model results are presented in the supplement for all cognitive measures (see Supplemental Tables S7 and S8 and Supplemental Figures 1, 2, and 3). While no significant moderation of sleep duration was observed on A_p_ or A_L_, the general patterns were consistent with prior findings [28], whereby genetic contributions to cognitive performance tended to decrease across increasing levels of sleep duration across most of the cognitive tasks. Examinations of these associations with the AD-PGS, excluding the *APOE* region (see Supplemental Tables S9 and S10), revealed similar patterns, though the results were not statistically significant.

#### Sleep disturbances

**Figure 3:**
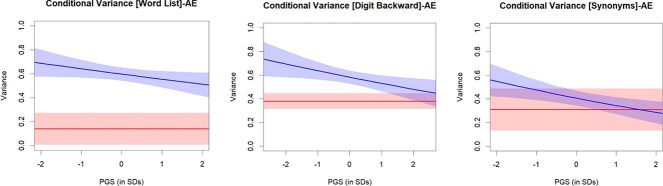
Genetic and environmental variance of word list, digit backward, and synonyms across varying levels of the AD-PGS. *Note.* Red lines = latent additive genetic variance A_L_, blue lines = nonshared environmental variance E. Conditional plots were generated from the fitted AE model by computing model-implied additive genetic (A_L_) and unique environmental (E) contributions across values of the measured A_P_ indexed by the AD polygenic score (AD-PGS; expressed in SD units), with other moderators fixed at their mean (0). Shading depicts 95% confidence intervals. These plots illustrate how model-implied effects change across the range of the AD-PGS.

For Word List, the AD-PGS accounted for a small but statistically significant proportion of variance (0.16%; B = –0.04, 95% CI = –0.07, –0.01; see Table 4) and significant moderation via the AD-PGS was observed for the E component (B = –0.03, 95% CI = –0.05, –0.003, FDR-corrected *p* = 0.049, see Table 4), suggesting significant environment-by-PGS interaction. The estimates suggest an attenuation of the contributions from the unique environmental influences with increasing levels of genetic risk for AD (see Figure 3). This attenuation suggests that in individuals with higher AD polygenic risk, the variability in episodic memory attributable to unique environmental experiences is reduced. In other words, genetic predisposition may exert a stronger and more constraining effect on episodic memory, limiting the degree to which environmental factors may influence cognitive outcomes. No significant moderation via sleep disturbance was observed on the AD-PGS (A_p_), nor for A_L_, C or E (see Table 4 for AE models and Supplemental Table S11 for ACE models).^2^ In examining these associations with the AD-PGS without the *APOE* region (see Supplemental Tables S12 and S13), the variance explained by A_p_ was small and non-significant (B = –0.003, 95% CI = –0.03, 0.03), and we no longer observed significant moderation of the AD-PGS on the E component.

For Animal Naming, the variance explained by the AD-PGS was small and nonsignificant (B = –0.03, 95% CI = –0.06, 0.01; see Table 4). Moreover, no significant moderation via sleep disturbance was observed on A_p_, A_L_, or E contributions in the AE models, nor on the C contributions in the fuller ACE models (see Supplemental Table S11). For the AD-PGS without the *APOE* region (see Supplemental Tables S12 and S13), no significant moderation was observed for sleep disturbance.

For Digits Forward, the variance explained by the AD-PGS was nonsignificant (B = 0.002, 95% CI = –0.03, 0.04; see Table 4). No significant moderation via the AD-PGS was observed on the E contributions to differences in Digits Forward. Moreover, sleep disturbance did not significantly moderate the A_p_, A_L_, or E components contributing to differences in performance. Examinations of the AD-PGS without the *APOE* region suggest the proportion of variance explained by the AD-PGS was small but significant (0.15%; B = –0.04, 95% CI = –0.07, –0.002); however, sleep disturbance did not significantly moderate the AD-PGS without the *APOE* region, A_L_ or E components in the AE model see Supplemental Tables S12 and S13).

For Digits Backward, the variance explained by the AD-PGS was small and nonsignificant (B = 0.02, 95% CI = –0.02, 0.05; see Table 4). However, a significant environment-by-PGS interaction was observed on the E component (B = –0.03, 95% CI = –0.05, –0.001, FDR-corrected *p* = 0.13, see Table 4) suggesting a reduction in the influences from the non-shared environment on working memory performance at higher levels of risk for AD (see Figure 3). As such, at higher levels of AD polygenic risk, the influence of non-shared environmental factors on working memory performance diminishes. Consequently, those with greater genetic risk may exhibit less environmental plasticity in working memory, meaning that environmental influences have a reduced impact on cognitive outcomes within this domain. However, this moderation effect was no longer significant after correction for multiple testing. In examinations of these associations with the AD-PGS without the *APOE* region, significant moderation of the E component was no longer observed (see Supplemental Tables S12 and S13).

For Synonyms, the AD-PGS, on its own, was not significant (B = –0.02, 95% CI = –0.08, 0.03) and explained very little of the variance in Synonyms performance (see Table 4). However, a significant AD-PGS moderation was observed on the E component (B = –0.05, 95% CI = –0.08, –0.02, FDR-corrected *p* = 0.01, see Table 4), suggesting a decrease in the contributions from the non-shared environmental influences on verbal ability performance with higher genetic risk for AD (see Figure 3). Like the other cognitive domains, individuals with greater AD polygenic risk exhibited diminished variability in verbal ability attributable to unique environmental experiences. No significant moderation of any component via sleep disturbance was observed. In examinations of these associations with the AD-PGS without the *APOE* region, significant moderation of the E component was no longer observed (see Supplemental Tables S12 and S13).

For Symbol Digit, all models with sleep disturbance as a moderator, in addition to the AD-PGS and age and showed no significant moderation of A_L_ or E for either the AD-PGS or sleep disturbance (see Table 4). In addition, within these models, sleep did not significantly moderate the portion of the genetic variance indexed by the AD-PGS on Symbol Digit performance. The variance explained by the AD-PGS was negligible and nonsignificant (B = –0.03, 95% CI = –0.07, 0.01). Similar results were observed for the AD-PGS without the *APOE* region (see Supplemental Tables S12 and S13).

## Discussion

The present study explored how underlying genetic and environmental contributions to cognition may be associated with poor sleep conditions, including shorter duration and increased disturbance, and interactions with genetic risk for AD. Across the six cognitive measures, the AD-PGS explained little direct variance in cognition, except for episodic memory (with the *APOE* region) and attention (without the *APOE* region). Moreover, the main effect of the AD-PGS did not vary meaningfully by sleep duration or sleep disturbance. Notably, environment-by-PGS interactions were observed for episodic memory, working memory, and verbal ability, where higher genetic risk for AD (full AD-PGS including *APOE*) was associated with reduced effects of individual-specific environmental influences. These findings suggest that individuals at higher genetic risk for AD may exhibit differential responsiveness to environmental influences on cognition, consistent with a gene–environment interaction framework. In other words, the effects of unique environmental factors on cognition may be constrained by genetic liability for AD. However, the observed environment-by-PGS interactions were modest, suggesting that while genetic risk may subtly constrain environmental influences on cognition, the overall impact is small. These small effect sizes underscore that, although patterns of gene–environment interplay are detectable, individual differences in cognition may be influenced by many other factors beyond sleep and AD polygenic risk.

Although prior research implicates sleep disturbances in synaptic plasticity deficits, impaired glymphatic clearance, AD pathology, and altered brain connectivity, the moderation of genetic influences on cognition by sleep disturbances was minimal in this study [51–73]. While general patterns suggested slightly higher genetic influences in poorer sleep contexts, consistent with our prior work [28], these effects did not reach statistical significance when both sleep and the AD-PGS were included as simultaneous moderators. Several factors may explain this limited moderation. First, the binary coding of sleep disturbance items indexing the sleep disturbance factor and the cross-sectional design may have reduced sensitivity to detect nuanced interactions. Second, sleep disturbances in the normative range captured in this sample may not constitute a sufficiently potent environmental stressor to amplify genetic influences on cognition, particularly in mid- to late-life adults. Biologically, while sleep disruption can impair synaptic homeostasis, hinder glymphatic clearance of neurotoxic waste such as β-amyloid, and alter connectivity within neural networks supporting cognition, these mechanisms may exert only subtle effects unless sleep disruption is chronic or severe. Third, the moderating effect of sleep may be context dependent. Although the AD-PGS accounted for a negligible proportion of total genetic variance, the variance in cognition explained by sleep may be overshadowed when polygenic risk is explicitly modeled. In other words, sleep may still act as an environmental “stressor,” but its detectable moderation effect is reduced in the presence of measured genetic risk for AD. While sleep disturbances alone showed minimal moderation, the inclusion of the AD-PGS revealed a broader picture of gene–environment interplay. Although the AD-PGS accounted for only a small proportion of overall genetic variance in cognitive performance, explaining less than 0.2% of the variance in any measure, it still meaningfully shaped the extent to which environmental factors influenced cognition.

Importantly, our findings demonstrate a gene–environment interaction in which higher AD-PGS was associated with reduced nonshared environmental contributions to cognition, particularly for episodic memory, working memory, and verbal ability. Among individuals with higher genetic risk for AD, nonshared environmental factors (e.g. unique person-specific lifestyle exposures) had smaller effects on cognition, indicating that genetic liability may limit environmental influence. While environmental effects may be overshadowed by genetic risk, the environment-by-PGS interactions detected were modest, suggesting that their practical impact on cognitive performance is small. Targeted interventions or enriched environments could still impact cognitive outcomes, though likely to a lesser extent than in lower-risk individuals. Moreover, additional factors beyond measured genetic risk may contribute to differences in cognitive performance. It is noted that AD is multifactorial and complex in nature and is highly heritable [74]. Even in examinations of the heritable contributions to AD, the AD-PGS was shown to only contribute to roughly 10% of the risk of AD, with even lower contributions observed for the AD-PGS without the *APOE* region.

Our findings further underscore the importance of considering both locus-specific and polygenic influences. We observed that the moderation of environmental influences on cognition by the AD-PGS was significant when the *APOE* region was included, but these effects disappeared once the *APOE* region was excluded, suggesting that *APOE* plays the predominant role. Adding *APOE* genotype (ε2, ε4) information to models of AD risk boosts prediction beyond an AD-PGS omitting the *APOE* region and is comparable to the full AD-PGS [41, 67]. Results from GWAS support *APOE* as the susceptibility loci for late-onset AD, specifically the ε4 allele [75]. The *APOE* ε4 allele has been consistently linked to poorer cognitive performance and accelerated cognitive decline [76, 77], affecting both global cognitive function [78] and various cognitive domains including memory and executive function [79–83]. Taken together, cognitive differences among high-risk individuals are thus largely genetically driven, and the impact of environmental exposures may be moderated by underlying genetic susceptibility for AD. These patterns are broadly consistent with present work that suggests a less pronounced effect from environmental factors on cognition (e.g. spatial-reasoning, semantic memory, episodic memory) among *APOE* ε4+ carriers, or carriers of putative risk alleles, compared to non-carriers [84, 85]. Overall, these findings underscore the importance of gene–environment interplay in understanding cognitive modifiability. While individuals at high genetic risk for AD may experience attenuated responsiveness to environmental influences on cognition, targeted strategies and interventions remain relevant. These findings provide empirical support for a GxE framework, suggesting that both genetic liability and environmental context shape cognitive outcomes, and inform efforts to optimize interventions based on underlying risk profiles to optimize effectiveness.

### Limitations

Several limitations should be acknowledged. First, the cross-sectional design restricts the ability to draw definitive conclusions about how sleep and AD-PGS moderate etiological contributions to cognition across time. Second, the study was underpowered relative to recent simulations [19], meaning that effect size estimates should be interpreted cautiously. Nevertheless, the detection of several moderation effects, even after FDR correction, provides valuable preliminary evidence. Consequently, some effects may have gone undetected, including replication of the statistically significant sleep duration moderation of additive genetic influences [28]. Additionally, the observed effect sizes for environment-by-PGS interactions were small, suggesting that while statistically detectable, their practical impact may be modest. Future studies leveraging larger samples will be essential to replicate and more precisely estimate these effects. Third, the sample was drawn from multiple studies, and harmonization of cognitive and sleep measures may potentially obscure some associations. Reliance on a simplified binary categorization of sleep disturbance items to define the factor scores and the use of single cognitive tests per domain may also have reduced sensitivity to effects. Finally, it must be noted that the *APOE* ε4 allele frequency is reduced with older age [86–88], where the hazard of death is greatly increased with increasing age due to conditions related to the deleterious effect of ε4 (e.g. cardiovascular disease, diabetes, AD) [87–89].

## Conclusion

Overall, this study examined the role of sleep, genetic risk for AD, and the varying etiological associations with cognition, leveraging the additional information gained from integrating a measured genetic factor into the classical twin model. While the AD-PGS showed limited contribution to genetic variance in the cognitive measures, we detected significant environment-by-PGS interactions. These interactions revealed that genetic risk was associated with varying responsiveness to environmental influences on cognitive performance. Our results suggest etiological heterogeneity, where individuals with a higher genetic predisposition to AD may experience a different set of risk factors compared to those with less genetic risk. However, since genetic risk accounts for only a portion of AD risk, and individuals with low genetic risk may still develop dementia, targeting modifiable environmental factors remains important. While we did not observe statistically significant moderation effects with sleep, our findings suggest a pattern whereby poorer sleep conditions, such as shorter duration and increased disturbances, may be associated with elevated genetic contributions to individual differences in cognitive performance. These results point to sleep as a potentially relevant factor in understanding cognitive aging and warrant further investigation in future studies. Additional work is needed to clarify these patterns.

## Supplementary Material

SLEEP_Supplementary_Materials_RR_v2_Submit_Final_zsag119

## Data Availability

IGEMS data are not publicly available given the variety of data agreements and regulations governing the different studies and countries. However, many of the individual studies participating in IGEMS do have ways to access their data, some by direct request to the participating study, and many of the datasets may be accessed through the National Archive of Computerized Data on Aging (NACDA). For the Swedish studies, see https://doi.org/10.3886/ICPSR03843.v2 (SATSA) and https://www.maelstromresearch.org/study/octo-twin (OCTO-Twin). For the United States study, see https://medschool.ucsd.edu/som/psychiatry/ research /VETSA/Researchers/Pages/default.aspx (VETSA). For access to data from the Danish Twin Registry, see https://www.sdu.dk/en/om_sdu/institutter_centre/ist_sundhedstjenesteforsk /centre/dtr/researcher (MADT). For the Australian study, see https://www.cheba.unsw.edu .au/research -projects/older-australian-twins-study (OATS).

## Footnotes

1 While the bivariate models followed a direct symmetric approach, the Bruins’ et al. model does not use the direct symmetric approach.

2 We note that the full ACE model was a better fitting model in the bivariate models; however, estimates from the full ACE models were consistent with findings from these more parsimonious AE models.

## Notes

**Institution where work was performed:** University of California, Riverside, University of California, San Diego, University of Colorado at Boulder.
