# Supplementary material for: Subjective sleep traits and cognition across mid- to late-adulthood: a cross-sectional study of gene–environment interplay
Source: Sleep. 2026 Apr 30;49(8):zsag119. doi: 10.1093/sleep/zsag119 (PMC13472895; doi:10.1093/sleep/zsag119)
Supplement: Supplementary file 1 [file sleep_supplementary_materials_rr_v2_submit_final_zsag119.docx]

**Title:** Subjective Sleep Traits and Cognition Across Mid- to Late-Adulthood: A Cross-sectional Study of Gene-Environment Interplay

FULL AUTHOR LIST:

Tina T. Vo-Eckerle^1-3^, Nathan A. Gillespie^4^, Kaare Christensen^5^, Deborah Finkel^6,7^, William S. Kremen^2,3^, Marianne Nygaard^5^, Perminder S. Sachdev^8^, Anbupalam Thalamuthu^8^, Chandra A. Reynolds^1,9,10^, for the IGEMS consortium.

AFFILIATIONS:

^1^ Department of Psychology, University of California, Riverside, Riverside, California, USA

^2^ Department of Psychiatry, University of California, San Diego, La Jolla, California, USA

^3^ Center for Behavior Genetics of Aging, University of California, San Diego, San Diego, California, USA

^4^Virginia Institute for Psychiatric and Behavioral Genetics, Virginia Commonwealth University, Richmond, Virginia, USA

^5^The Danish Twin Registry, Department of Public Health, University of Southern Denmark, Odense, Denmark

^6^Institute of Gerontology, School of Health and Welfare, Jönköping University, Jönköping, Sweden

^7^Center for Economic and Social Research, University of Southern California, Los Angeles, USA

^8^Centre for Healthy Brain Ageing (CHeBA), Discipline of Psychiatry and Mental Health, School of Clinical Medicine, UNSW, Sydney, Australia

^9^Institute for Behavioral Genetics, University of Colorado Boulder, Boulder, Colorado, USA

^10^Department of Psychology and Neuroscience, University of Colorado Boulder, Boulder, Colorado, USA

**Corresponding Author(s):** Tina T. Vo & Chandra A. Reynolds

**Email and Phone**: [ttv008@health.ucsd.edu](mailto:ttv008@health.ucsd.edu) (714-613-3707), chandra.reynolds@colorado.edu

**Corresponding Author Address:** 3252 Holiday Court, La Jolla, CA 92037

**Institution where work was performed:** University of California, Riverside, University of California, San Diego, University of Colorado at Boulder

| **Supplemental Table S1** Individual Sleep Disturbance Items from IGEMS | | |
| --- | --- | --- |
|  | Item | Response Options |
| *LSADT | *Wake_early*: "Do you wake up early in the morning without being able to fall asleep again?" | Original: 1= most of the time, 2= sometimes, 3= no  Recoded: 1,2= 1 some endorsement, 3= 0 no endorsement |
| *MADT | *Wake_early:* "Do you wake up early in the morning without being able to fall asleep again?" | Original: 1= most of the time, 2= sometimes, 3= no  Recoded: 1,2= 1 some endorsement, 3= 0 no endorsement |
| MIDUS | *Wake_early:* “Wake up too early frequency, please indicate how often you experience each of the following: wake up too early in the morning and be unable to get back to sleep” | Original: Never (0 times), rarely (once a month or less), sometimes (2-4 times per month), often (2-3 times per week), almost always (4 or more times per week)  Recoded: 1&2=no endorsement, 3-5=some endorsement |
| *SATSA | *Wake_early*: “Has it occurred to you that you have woke up too early and was not able to fall asleep?” | Original: 1=never, 2=rarely, 3= sometimes, 4= most often, 5=always  Recoded: 1&2=no endorsement, 3-5=some endorsement |
| *GENDER | *Wake_early*: “I wake up in the early hours of the morning” | Original: 0=no, 1=yes  Recoded: 0=No endorsement, 1=some endorsement |
| MIDUS | *Wake_night*: “Wake up during the night frequency, please indicate how often you experienced each of the following: wake up during the night and have difficulty going back to sleep” | Original: Never (0 times), rarely (once a month or less), sometimes (2-4 times per month), often (2-3 times per week), almost always (4 or more times per week)  Recoded: 1&2=no endorsement, 3-5=some endorsement |
| *VETSA | *Wake_night*: “Wake up in the middle of the night or early morning-trouble sleep” | Original: 1= not during the past month, 2= less than once a week, 3= once or twice a week, 4= three or more times a week  Recoded: 1&2=no endorsement, 3-5=some endorsement |
| *SATSA | *Wake_night* : “Have you woken up in the middle of the night and have had trouble falling asleep?” | Original: 1=never, 2=rarely, 3= sometimes, 4= most often, 5=always  Recoded: 1&2=no endorsement, 3-5=some endorsement |
| *VETSA | *Restless*: “Sleep was restless” | Original: 1=rarely or none of the time (less than 1 day), 2= some or little of the time (1-2 days), 3= occasionally or a moderate amount of time (3-4 days), 4= most or all of the time (5-7 days)  Recoded: 1&2=no endorsement, 3-4=some endorsement |
| MTSADA | *Restless:* “Sleep was restless” | Original: rarely or none of the time, some of the time, occasionally, most of the time  Recoded: 1&2=no endorsement, 3-4=some endorsement |
| *OCTO-Twin | *Restless:* “Sleep was restless” | Original: rarely or none of the time, some of the time, occasionally, most of the time  Recoded: 1&2=no endorsement, 3-4=some endorsement |
| OCTO-Gender | *Restless:* “Sleep was restless” | Original: rarely or none of the time, some of the time, occasionally, most of the time  Recoded: 1&2=no endorsement, 3-4=some endorsement |
| *SATSA | *Restless:* “Did not sleep well/Restless sleep” | Original: 1=Never/almost never, 2=rather seldom, 3=Quite often, 4= always/almost always  Recoded: 1&2=no endorsement, 3-4=some endorsement |
| *GENDER | *Restless:* “Slept poorly/restless sleep” | Original: 0=seldom or never, 1=rather seldom, 2=rather often, 3=almost always or always  Recoded: 1&2=no endorsement, 3-4=some endorsement |
| *SATSA | *Snore:* “Have you been severely snoring?” | Original: 1=never, 2=rarely, 3=sometimes, 4=most often, 5=always  Recoded: 1&2=no endorsement, 3-4=some endorsement |
| *VETSA | *Snore:* “Trouble sleep-snore, how often have you had trouble sleeping because you cough or snore loudly” | Original:1= not during the past month, 2= less than once a week, 3= once or twice a week  4= three or more times a week  Recoded: 1&2=no endorsement, 3-4=some endorsement |
| *OATS | *Snore:* “Do you snore” | Original:1=yes, 2=no  Recoded:2=no endorsement, 1=some endorsement |
| *VETSA | *Apnea*: “Have you ever been told by a physician that you had any of the following conditions or illnesses? - sleep apnea” | Original: 1=yes, 2=no  Recoded: 2= no endorsement, 1=some endorsement |
| *OATS | *Apnea*: “Diagnosed with sleep apnoea” | Original:1=no, 2=yes  Recoded:1=no endorsement, 2=some endorsement |
| MTSADA | *Nightmare:* “Do you have nightmares?”  Variable name: nightmare | Original: 1=no, 2=yes  Recoded: 1=no endorsement, 2=some endorsement |
| *SATSA | *Nightmare:* “Have you had nightmares?” | Original: 1=never, 2=rarely, 3=sometimes, 4=most often, 5=always  Recoded: 1&2=no endorsement, 3-4=some endorsement |
| *VETSA | *Nightmare:* “Trouble sleep- Have bad dreams” | Original: 1= not during the past month, 2= less than once a week, 3= once or twice a week, 4= three or more times a week  Recoded: 1&2=no endorsement, 3-4=some endorsement |
| *GENDER | *Medication:* “I take tablets to help me sleep” | Original: 0=No, 1=yes  Recoded: 0=No endorsement, 1=Some endorsement |
| *VETSA | *Medication:* “Taken meds to help sleep” | Original:1= no during the past month, 2= less than once a week, 3= once or twice a week  4= three or more times a week  Recoded: 1&2=no endorsement, 3-4=some endorsement |
| MIDUS | *Medication:* “Used sedatives on own ever (12 mo)” | Original: 1=yes, 2=no  Recoded: 2=no endorsement, 1=some endorsement |
| *SATSA | *Latency:* “Have you had problems falling asleep in the evening?” | Original: 1= never, 2=rarely, 3=sometimes, 4= most often, 5= always  Recoded: 1&2=no endorsement, 3-4=some endorsement |
| *GENDER | *Latency:* “It takes me a long time to get to sleep” | Original: 0=no, 1=yes  Recoded: 0=no endorsement, 1=some endorsement |
| *VETSA | *Latency:* “Trouble sleep- cannot sleep within 30 min” | Original: 1= No during the past month, 2= less than once a week, 3= once or twice a week, 4= three or more times a week  Recoded: 1&2=no endorsement, 3-4=some endorsement |
| MIDUS | *Latency:* “Trouble falling asleep frequency- (30 days)” | Original: 1= almost every day, 2= several times a week, 3= once a week, 4= several times a month, 5= once a month  Recoded: 1&2=no endorsement, 3-5=some endorsement |
| *OCTO-Twin | *Latency:* “Difficulty sleeping” | Original: 0=no, 1=yes  Recoded: 0= no endorsement, 1= some endorsement |
| *OATS | *Latency:* “Difficulty falling asleep” | Original: 1=yes, 2=no  Recoded: 2=no endorsement, 1=some endorsement |
| OCTO-Gender | *Latency:* “Difficulty sleeping” | Original: 0=no, 1=yes  Recoded: 0= no endorsement, 1= some endorsement |

*Note. ** = Sample was utilized in the present analyses. SATSA= Swedish Adoption/Twin Study of Aging, OCTO-Twin= Origins of Variance in the Oldest Old, GENDER= Aging in Women and Men: A Longitudinal Study of Gender Differences in Health Behavior and Health among Elderly, OCTO-Gender=Older GENDER participants who followed the OCTO-Twin protocol, MIDUS= Midlife in the United States, MTSADA= Minnesota Twin Study of Adult Development and Aging, VETSA= Vietnam Era Twin Study of Aging, MADT=Middle Age Danish Twins Study, LSADT=Longitudinal Study of Aging Danish Twins, OATS= Older Australian Twin Study. All available IGEMS studies with sleep disturbance data contributed to sleep disturbance harmonization and factor score generation. However, not all studies presented in this table were included in the present biometrical model analyses as not all IGEMS studies had scores for the Alzheimer’s disease polygenic score.

**Supplemental Table S2** Factor Loadings and Model Fit Statistics for Confirmatory Factor Analysis of Sleep Disturbances

|  | Model 1  (One Factor CFA) | | Model 2  (Two Factor CFA)  FS1 | | Model 2  (Two Factor CFA)  FS2 | |
| --- | --- | --- | --- | --- | --- | --- |
| Item | Estimate | SE | Estimate | SE | Estimate | SE |
| Wake_early | 0.83 | 0.03 | 0.83 | 0.03 | -- | -- |
| Wake_night | 0.75 | 0.03 | 0.75 | 0.03 | -- | -- |
| Snore* | 0.35 | 0.04 | -- | -- | 0.62 | 0.12 |
| Medication Usage | 0.52 | 0.04 | 0.52 | 0.04 | -- | -- |
| Restless | 0.78 | 0.03 | 0.78 | 0.03 | -- | -- |
| Latency | 0.62 | 0.02 | 0.62 | 0.02 | -- | -- |
| Apnea* | 0.29 | 0.07 | -- | -- | 0.42 | 0.09 |
| Nightmare | 0.65 | 0.04 | 0.65 | 0.04 | -- | -- |
| Model Fit Statistics |  |  |  |  |  |  |
| AIC | 27791.643 | | 27785.875 | | | |
| BIC | 27909.933 | | 27911.559 | | | |
| Sample-Size Adjusted BIC | 27859.087 | | 27857.535 | | | |
| Number of Free Parameters | 16 | | 17 | | | |
| Correlation FS1 & FS2 | -- | | .94 | | | |

*Note.* CFA=Confirmatory Factor Analysis, Model 1= One-factor CFA with separate factor loadings for each item category (wake_early, wake_night, snore, sleep medication, restless, latency, apnea, nightmare). Model 2= Two-factor CFA with separate factor loadings where the first factor reflected sleep efficiency (wake_early, wake_night, medication usage, restless, latency, nightmare) and the second factor reflected breathing-related sleep disturbances (snore, apnea). *= Items on the second factor. Despite Model 2 providing a more optimal model fit, we proceeded with Model 1 as both factors in Model 2 were highly correlated and the sample size was large (*N=*12006).

**Supplemental Table S3** Univariate ACE Estimates by Country

|  | VA | VC | VE |
| --- | --- | --- | --- |
| ***Sleep Duration*** |  |  |  |
| Full Sample | 0.13 [-0.05, 0.31] | 0.24 [0.09, 0.38] | 0.63 [0.57, 0.70] |
|  | 0.40 [0.35, 0.46] | -- | 0.60 [0.54, 0.65] |
| Sweden | 0.57 [-0.11, 1.39] | -0.04 [-0.80, 0.52] | 0.47 [0.31, 0.69] |
|  | 0.53 [0.32, 0.68] | -- | 0.47 [0.32, 0.68] |
| United States | 0.27 [-0.05, 0.59] | 0.01 [-0.27, 0.27] | 0.72 [0.63, 0.83] |
|  | 0.28 [0.18, 0.37] | -- | 0.72 [0.63, 0.82] |
| Denmark | 0.35 [0.07, 0.63] | -0.08 [-0.30, 0.12] | 0.74 [0.64, 0.85] |
|  | 0.24 [0.15, 0.33] | -- | 0.76 [0.67, 0.85] |
| Australia | -- | -- | -- |
|  | -- | -- | -- |
| ***Sleep Disturbances*** |  |  |  |
| Full Sample | 0.05 [-0.12, 0.22] | 0.17 [0.04, 0.30] | 0.78 [0.72, 0.84] |
|  | 0.26 [0.20, 0.31] | -- | 0.74 [0.69, 0.80] |
| Sweden | -0.30 [-0.84, 0.20] | 0.43 [0.10, 0.77] | 0.87 [0.65, 1.11] |
|  | 0.31 [0.14, 0.45] | -- | 0.69 [0.55, 0.86] |
| United States | 0.22 [-0.10, 0.54] | 0.01 [-0.26, 0.27] | 0.77 [0.67, 0.88] |
|  | 0.23 [0.13, 0.33] | -- | 0.77 [0.67, 0.87] |
| Denmark | 0.15 [-0.12, 0.41] | -0.01 [-0.21, 0.19] | 0.86 [0.76, 0.97] |
|  | 0.13 [0.04, 0.22] | -- | 0.87 [0.78, 0.96] |
| Australia | 0.09 [-0.38, 0.58] | 0.13 [-0.29, 0.51] | 0.78 [0.63, 0.94] |
|  | 0.24 [0.09, 0.37] | -- | 0.76 [0.63, 0.91] |
| ***Word List*** |  |  |  |
| Full Sample | 0.23 [0.07, 0.38] | 0.17 [0.05, 0.29] | 0.60 [0.55, 0.66] |
|  | 0.43 [0.38, 0.48] | -- | 0.57 [0.52, 0.62] |
| Sweden | 0.73 [0.14, 1.16] | -0.16 [-0.48, 0.19] | 0.44 [0.27, 0.71] |
|  | 0.49 [0.29, 0.65] | -- | 0.51 [0.35, 0.71] |
| United States | -0.11 [0.42, 0.21] | 0.31 [0.05, 0.55] | 0.80 [0.70, 0.92] |
|  | 0.24 [0.15, 0.34] | -- | 0.76 [0.66, 0.85] |
| Denmark | 0.24 [0.01, 0.45] | 0.18 [0.0003, 0.35] | 0.59 [0.51, 0.67] |
|  | 0.44 [0.37, 0.51] | -- | 0.56 [0.49, 0.63] |
| Australia | 0.16 [-0.30, 0.65] | 0.15 [-0.27, 0.53] | 0.69 [0.55, 0.84] |
|  | 0.32 [0.19, 0.46] | -- | 0.67 [0.54, 0.81] |
| ***Animal Naming*** |  |  |  |
| Full Sample | 0.41 [0.25, 0.58] | 0.07 [-0.07, 0.20] | 0.52 [0.47, 0.58] |
|  | 0.49 [0.43, 0.54] | -- | 0.51 [0.46, 0.57] |
| Sweden | -- | -- | -- |
|  | -- | -- | -- |
| United States | 0.61 [0.32, 0.90] | -0.16 [-0.42, 0.07] | 0.56 [0.47, 0.66] |
|  | 0.42 [0.32, 0.50] | -- | 0.58 [0.50, 0.68] |
| Denmark | 0.33 [0.12, 0.53] | 0.17 [0.001, 0.32] | 0.50 [0.43, 0.58] |
|  | 0.53 [0.46, 0.59] | -- | 0.47 [0.41, 0.54] |
| Australia | -- | -- | -- |
|  | -- | -- | -- |
| ***Digit Forward*** |  |  |  |
| Full Sample | 0.38[ 0.22, 0.53] | 0.09 [-0.03, 0.22] | 0.53 [0.48, 0.58] |
|  | 0.48 [0.44, 0.53] | -- | 0.52 [0.47, 0.56] |
| Sweden | 0.68 [0.01, 1.30] | -0.17 [-0.70, 0.32] | 0.49 [0.32, 0.75] |
|  | 0.48 [0.24, 0.66] | -- | 0.52 [0.34, 0.76] |
| United States | 0.47 [0.21, 0.76] | 0.04 [-0.22, 0.27] | 0.49 [0.42, 0.57] |
|  | 0.39 [0.30, 0.47] | -- | 0.61 [0.53, 0.70] |
| Denmark | 0.22 [-0.01, 0.45] | 0.15 [-0.03, 0.33] | 0.62 [0.54, 0.71] |
|  | 0.41 [0.33, 0.48] | -- | 0.59 [0.52, 0.67] |
| Australia | 0.46 [0.06, 0.90] | 0.02 [-0.37, 0.36] | 0.52 [0.41, 0.65] |
|  | 0.49 [0.36, 0.59] | -- | 0.51 [0.41, 0.64] |
| ***Digit Backward*** |  |  |  |
| Full Sample | 0.40 [0.24, 0.55] | 0.05 [-0.08, 0.17] | 0.56 [0.51, 0.61] |
|  | 0.45 [0.40, 0.50] | -- | 0.55 [0.50, 0.60] |
| Sweden | 1.59 [0.93, 2.15] | -0.98 [-1.46, -0.47] | 0.39 [0.25, 0.64] |
|  | 0.32 [-0.01, 0.61] | -- | 0.68 [0.39, 1.01] |
| United States | 0.60 [0.29, 0.92] | -0.19 [-0.47, 0.07] | 0.59 [0.51, 0.68] |
|  | 0.39 [0.30, 0.47] | -- | 0.61 [0.53, 0.70] |
| Denmark | 0.16 [-0.08, 0.38] | 0.23 [0.05, 0.39] | 0.62 [0.53, 0.71] |
|  | 0.43 [0.36, 0.50] | -- | 0.57 [0.50, 0.64] |
| Australia | 0.52 [0.13, 0.94] | 0.03 [-0.35, 0.36] | 0.45 [0.35, 0.57] |
|  | 0.55 [0.44, 0.65]] | -- | 0.45 [0.35, 0.56] |
| ***Synonyms*** |  |  |  |
| Full Sample | 0.62 [0.45, 0.79] | 0.06 [-0.09, 0.20] | 0.32 [0.27, 0.39] |
|  | 0.68 [0.63, 0.73] | -- | 0.32 [0.27, 0.37] |
| Sweden | 0.56 [019, 0.86] | 0.15 [-0.10, 0.39] | 0.29 [0.20, 0.47] |
|  | 0.73 [0.62, 0.81] | -- | 0.27 [0.19, 0.38] |
| United States | 0.42 [0.14, 0.74] | 0.01 [-0.28, 0.27] | 0.57 [0.49, 0.67] |
|  | 0.43 [0.35, 0.51] | -- | 0.57 [0.49, 0.65] |
| Denmark | -- | -- | -- |
|  | -- | -- | -- |
| Australia | -- | -- | -- |
|  | -- | -- | -- |
| ***Symbol Digit*** |  |  |  |
| Full Sample | 0.43 [0.30, 0.56] | 0.29 [0.17, 0.40] | 0.28 [0.25, 0.33] |
|  | 0.73 [0.69, 0.76] | -- | 0.27 [0.24, 0.31] |
| Sweden | 0.43 [0.09, 0.75] | 0.28 [-0.004, 0.53] | 0.29 [0.20, 0.43] |
|  | 0.73 [0.62, 0.81] | -- | 0.27 [0.19, 0.38] |
| United States | -- | -- | -- |
|  | -- | -- | -- |
| Denmark | 0.46 [0.26, 0.66] | 0.18 [0.01, 0.34] | 0.36 [0.30, 0.44] |
|  | 0.66 [0.60, 0.71] | -- | 0.34 [0.29, 0.40] |
| Australia | 0.62 [0.30, 0.99] | 0.07 [-0.28, 0.35] | 0.31 [0.24, 0.41] |
|  | 0.69 [0.60, 0.76] | -- | 0.31 [0.24, 0.40] |

**Supplemental Table S4** Fit Statistics for Bivariate ACE Models

|  | Models | K | | -2LL | | df | | AIC | | Δχ^2^ | | *Δdf* | | *p* | |  |
| --- | --- | --- | --- | --- | --- | --- | --- | --- | --- | --- | --- | --- | --- | --- | --- | --- |
| Word List | 1. ACE (Sleep Duration and Word List) | 15 | | 27565.80 | | 5390 | | 27595.80 | |  | |  | |  | |  |
|  | 2. AE (Sleep Duration and Word List) | 12 | | 27572.99 | | 5393 | | 27596.99 | | 7.19 | | 3 | | 0.066 | |  |
|  | 3. CE (Sleep Duration and Word List) | 12 | | 27575.21 | | 5393 | | 27599.21 | | 9.41 | | 3 | | 0.024 | |  |
|  | 4. E (Sleep Duration and Word List) | 9 | | 27750.24 | | 5396 | | 27768.24 | | 184.44 | | 6 | | 3.87E-37 | |  |
|  | 5. Drop A21, C21, E21 | 12 | | 27570.43 | | 5393 | | 27594.43 | | 4.63 | | 3 | | 0.201 | |  |
|  | 1. ACE (Sleep Disturbances and Word List) | 15 | | 39739.91 | | 7680 | | 39769.91 | |  | |  | |  | |  |
|  | 2. AE (Sleep Disturbances and Word List) | 12 | | 39754.24 | | 7683 | | 39778.24 | | 14.33 | | 3 | | 0.002 | |  |
|  | 3. CE (Sleep Disturbances and Word List) | 12 | | 39743.40 | | 7683 | | 39767.40 | | 3.49 | | 3 | | 0.322 | |  |
|  | 4. E (Sleep Disturbances and Word List) | 9 | | 39976.68 | | 7686 | | 39994.68 | | 236.77 | | 6 | | 2.75E-48 | |  |
|  | 5. Drop A21, C21, E21 | 12 | | 39755.40 | | 7683 | | 39779.40 | | 15.49 | | 3 | | 0.001 | |  |
| Animal Naming | 1. ACE (Sleep Duration and Animal Naming) | 15 | | 26334.08 | | 5252 | | 26364.08 | |  | |  | |  | |  |
|  | 2. AE (Sleep Duration and Animal Naming) | 12 | | 26336.10 | | 5255 | | 26360.10 | | 2.02 | | 3 | | 0.569 | |  |
|  | 3. CE (Sleep Duration and Animal Naming) | 12 | | 26362.69 | | 5255 | | 26386.69 | | 28.61 | | 3 | | 2.71E-06 | |  |
|  | 4. E (Sleep Duration and Animal Naming) | 9 | | 26556.34 | | 5258 | | 26574.34 | | 222.25 | | 6 | | 3.44E-45 | |  |
|  | 5. Drop A21, C21, E21 | 12 | | 26336.72 | | 5255 | | 26360.72 | | 2.64 | | 3 | | 0.451 | |  |
|  | 1. ACE (Sleep Disturbances and Animal Naming) | 15 | | 31968.41 | | 6710 | | 31998.41 | |  | |  | |  | |  |
|  | 2. AE (Sleep Disturbances and Animal Naming) | 12 | | 31973.85 | | 6713 | | 31997.85 | | 5.44 | | 3 | | 0.143 | |  |
|  | 3. CE (Sleep Disturbances and Animal Naming) | 12 | | 31991.40 | | 6713 | | 32015.40 | | 22.99 | | 3 | | 4.07E-05 | |  |
|  | 4. E (Sleep Disturbances and Animal Naming) | 9 | | 32187.08 | | 6716 | | 32205.08 | | 218.67 | | 6 | | 2.00E-44 | |  |
|  | 5. Drop A21, C21, E21 | 12 | | 31972.74 | | 6713 | | 31996.74 | | 4.32 | | 3 | | 0.229 | |  |
| Digits Forward | 1. ACE (Sleep Duration and Digits Forward) | | 15 | | 28140.73 | | 5434 | | 28170.73 | |  | |  | |  | |
|  | 2. AE (Sleep Duration and Digits Forward) | | 12 | | 28144.94 | | 5437 | | 28168.94 | | 4.21 | | 3 | | 0.239 | |
|  | 3. CE (Sleep Duration and Digits Forward) | | 12 | | 28164.85 | | 5437 | | 28188.85 | | 24.12 | | 3 | | 2.36E-05 | |
|  | 4. E (Sleep Duration and Digits Forward) | | 9 | | 28444.85 | | 5440 | | 28462.85 | | 304.12 | | 6 | | 1.07E-62 | |
|  | 5. Drop A21, C21, E21 | | 12 | | 28143.47 | | 5437 | | 28167.47 | | 2.74 | | 3 | | 0.433 | |
|  | 1. ACE (Sleep Disturbances and Digits Forward) | | 15 | | 37617.88 | | 7407 | | 37647.88 | |  | |  | |  | |
|  | 2. AE (Sleep Disturbances and Digits Forward) | | 12 | | 37624.93 | | 7410 | | 37648.93 | | 7.05 | | 3 | | 0.070 | |
|  | 3. CE (Sleep Disturbances and Digits Forward) | | 12 | | 37641.79 | | 7410 | | 37665.79 | | 23.92 | | 3 | | 2.60E-05 | |
|  | 4. E (Sleep Disturbances and Digits Forward) | | 9 | | 37963.15 | | 7413 | | 37981.15 | | 345.28 | | 6 | | 1.59E-71 | |
|  | 5. Drop A21, C21, E21 | | 12 | | 37627.05 | | 7410 | | 37651.05 | | 9.17 | | 3 | | 0.027 | |
|  | 1. ACE (Sleep Duration and Digits Backward) | | 15 | | 28097.56 | | 5433 | | 28127.56 | |  | |  | |  | |
| Digits Backward | 2. AE (Sleep Duration and Digits Backward) | | 12 | | 28099.51 | | 5436 | | 28123.51 | | 1.95 | | 3 | | 0.583 | |
|  | 3. CE (Sleep Duration and Digits Backward) | | 12 | | 28125.04 | | 5436 | | 28149.04 | | 27.48 | | 3 | | 4.67E-06 | |
|  | 4. E (Sleep Duration and Digits Backward) | | 9 | | 28347.79 | | 5439 | | 28365.79 | | 250.23 | | 6 | | 3.66E-51 | |
|  | 5. Drop A21, C21, E21 | | 12 | | 28100.59 | | 5436 | | 28124.59 | | 3.03 | | 3 | | 0.387 | |
|  | 1. ACE (Sleep Disturbances and Digits Backward) | | 15 | | 37512.79 | | 7404 | | 37542.79 | |  | |  | |  | |
|  | 2. AE (Sleep Disturbances and Digits Backward) | | 12 | | 37518.16 | | 7407 | | 37542.16 | | 5.38 | | 3 | | 0.146 | |
|  | 3. CE (Sleep Disturbances and Digits Backward) | | 12 | | 37540.58 | | 7407 | | 37564.58 | | 27.79 | | 3 | | 4.02E-06 | |
|  | 4. E (Sleep Disturbances and Digits Backward) | | 9 | | 37807.19 | | 7410 | | 37825.19 | | 294.40 | | 6 | | 1.29e-60 | |
|  | 5. Drop A21, C21, E21 | | 12 | | 37527.12 | | 7407 | | 37551.12 | | 14.33 | | 3 | | 0.002 | |
| Synonyms | 1. ACE (Sleep Duration and Synonyms) | 15 | | 155633.31 | | 3975 | | 15593.31 | |  | |  | |  | |  |
|  | 2. AE (Sleep Duration and Synonyms) | 12 | | 15567.58 | | 3978 | | 15591.58 | | 4.27 | | 3 | | 0.234 | |  |
|  | 3. CE (Sleep Duration and Synonyms) | 12 | | 15593.12 | | 3978 | | 15617.12 | | 29.81 | | 3 | | 1.51E-06 | |  |
|  | 4. E (Sleep Duration and Synonyms) | 9 | | 15801.61 | | 3981 | | 15819.61 | | 238.30 | | 6 | | 1.30E-48 | |  |
|  | 5. Drop A21, C21, E21 | 12 | | 15568.33 | | 3978 | | 15592.33 | | 5.02 | | 3 | | 0.170 | |  |
|  | 1. ACE (Sleep Disturbances and Synonyms) | 15 | | 22201.83 | | 5528 | | 22231.83 | |  | |  | |  | |  |
|  | 2. AE (Sleep Disturbances and Synonyms) | 12 | | 22207.39 | | 5531 | | 22231.39 | | 5.56 | | 3 | | 0.135 | |  |
|  | 3. CE (Sleep Disturbances and Synonyms) | 12 | | 22242.02 | | 5531 | | 22266.02 | | 40.19 | | 3 | | 9.71E-09 | |  |
|  | 4. E (Sleep Disturbances and Synonyms) | 9 | | 22487.50 | | 5534 | | 22505.50 | | 285.67 | | 6 | | 9.59E-59 | |  |
|  | 5. Drop A21, C21, E21 | 12 | | 22206.11 | | 5531 | | 22230.11 | | 4.28 | | 3 | | 0.233 | |  |
| Symbol Digit | 1. ACE (Sleep Duration and Symbol Digit) | 15 | | 18677.75 | | 4272 | | 18707.45 | |  | |  | |  | |  |
|  | 2. AE (Sleep Duration and Symbol Digit) | 12 | | 18680.34 | | 4275 | | 18704.34 | | 2.60 | | 3 | | 0.458 | |  |
|  | 3. CE (Sleep Duration and Symbol Digit) | 12 | | 18709.50 | | 4275 | | 18733.50 | | 31.75 | | 3 | | 5.91E-07 | |  |
|  | 4. E (Sleep Duration and Symbol Digit) | 9 | | 18987.21 | | 4278 | | 19005.21 | | 309.46 | | 6 | | 7.66E-64 | |  |
|  | 5. Drop A21, C21, E21 | 12 | | 18678.77 | | 4275 | | 18702.77 | | 1.03 | | 3 | | 0.794 | |  |
|  | 1. ACE (Sleep Disturbances and Symbol Digit) | 15 | | 27986.48 | | 6232 | | 28016.48 | |  | |  | |  | |  |
|  | 2. AE (Sleep Disturbances and Symbol Digit) | 12 | | 27994.68 | | 6235 | | 28018.68 | | 8.21 | | 3 | | 0.042 | |  |
|  | 3. CE (Sleep Disturbances and Symbol Digit) | 12 | | 28027.08 | | 6235 | | 28051.08 | | 40.60 | | 3 | | 7.95E-09 | |  |
|  | 4. E (Sleep Disturbances and Symbol Digit) | 9 | | 28384.01 | | 6238 | | 28402.01 | | 397.53 | | 6 | | 9.48E-83 | |  |
|  | 5. Drop A21, C21, E21 | 12 | | 27988.99 | | 6235 | | 28012.99 | | 2.51 | | 3 | | 0.473 | |  |

*Note*. A=additive genetic influences, C=shared environmental influences, E=non-shared environmental influences. A21, C21, E21= genetic and environmental influences shared between sleep and cognition.

**Supplemental Table S5** Fit Statistics for Moderation Models

| Models | K | 2LL | df | AIC | Δχ^2^ | *Δdf* | *p* |
| --- | --- | --- | --- | --- | --- | --- | --- |
| *Word List (PGS, Sleep Duration, Age)* | | | | | | | |
| 1. ACE | 23 | 13049.2 | 4706 | 13095.2 |  |  |  |
| 2. AE | 19 | 13058.7 | 4710 | 13096.7 | 9.51 | 4 | 0.05 |
| 3. CE | 17 | 13059.0 | 4712 | 13093.0 | 9.79 | 6 | 0.13 |
| 4. E | 13 | 13132.8 | 4716 | 13158.8 | 83.61 | 10 | 9.82E-14 |
| *Word List (PGS, Sleep Disturbances, Age)* | | | | | | | |
| 1. ACE | 24 | 17989.3 | 6700 | 18037.3 |  |  |  |
| 2. AE | 20 | 17992.4 | 6704 | 18032.4 | 3.16 | 4 | 0.53 |
| 3. CE | 18 | 18006.5 | 6706 | 18042.5 | 17.21 | 6 | 0.01 |
| 4. E | 14 | 18096.1 | 6710 | 18124.1 | 106.79 | 10 | 2.37E-18 |
| *Animal Naming (PGS, Sleep Duration, Age)* | | | | | | | |
| 1. ACE | 22 | 12369.4 | 4452 | 12413.4 |  |  |  |
| 2. AE | 18 | 12372.0 | 4456 | 12408.0 | 2.62 | 4 | 0.62 |
| 3. CE | 16 | 12387.4 | 4458 | 12419.4 | 18.01 | 6 | 0.01 |
| 4. E | 12 | 12491.8 | 4462 | 12515.8 | 122.47 | 10 | 1.59E-21 |
| *Animal Naming (PGS, Sleep Disturbances, Age)* | | | | | | | |
| 1. ACE | 22 | 13481.9 | 4958 | 13525.9 |  |  |  |
| 2. AE | 18 | 13486.6 | 4962 | 13522.6 | 4.65 | 4 | 0.33 |
| 3. CE | 16 | 13509.0 | 4964 | 13541.0 | 27.09 | 6 | .0001 |
| 4. E | 12 | 13631.5 | 4968 | 13655.5 | 149.57 | 10 | 4.57E-27 |
| *Digit Forward (PGS, Sleep Duration, Age)* | | | | | | | |
| 1. ACE | 23 | 13210.7 | 4781 | 13256.7 |  |  |  |
| 2. AE | 19 | 13218.5 | 4785 | 13256.5 | 7.77 | 4 | 0.10 |
| 3. CE | 17 | 13238.7 | 4787 | 13272.7 | 27.99 | 6 | 9.44E-05 |
| 4. E | 13 | 13417.3 | 4791 | 13443.3 | 206.61 | 10 | 6.72E-39 |
| *Digit Forward (PGS, Sleep Disturbances, Age)* | | | | | | | |
| 1. ACE | 24 | 16906.1 | 6157 | 16954.1 |  |  |  |
| 2. AE | 20 | 16917.4 | 6161 | 16957.4 | 11.30 | 4 | 0.02 |
| 3. CE | 18 | 16944.9 | 6163 | 16980.9 | 38.79 | 6 | 7.86E-07 |
| 4. E | 14 | 17174.8 | 6167 | 17202.8 | 268.75 | 10 | 6.14E-52 |
| *Digit Backward (PGS, Sleep Duration, Age)* | | | | | | | |
| 1. ACE | 23 | 13209.8 | 4778 | 13255.8 |  |  |  |
| 2. AE | 19 | 13212.7 | 4782 | 13250.7 | 2.92 | 4 | 0.57 |
| 3. CE | 17 | 13224.0 | 4784 | 13258.0 | 14.21 | 6 | 0.03 |
| 4. E | 13 | 13357.2 | 4788 | 13383.2 | 147.38 | 10 | 1.29E-26 |
| *Digit Backward (PGS, Sleep Disturbances, Age)* | | | | | | | |
| 1. ACE | 24 | 16885.2 | 6152 | 16933.2 |  |  |  |
| 2. AE | 20 | 16913.7 | 6156 | 16953.7 | 28.56 | 4 | 9.61E-06 |
| 3. CE | 18 | 16934.2 | 6158 | 16970.2 | 49.08 | 6 | 7.20E-09 |
| 4. E | 14 | 17112.6 | 6162 | 17140.6 | 227.41 | 10 | 3.00E-43 |
| *Synonyms (PGS, Sleep Duration, Age)* | | | | | | | |
| 1. ACE | 22 | 5785.91 | 2150 | 5829.91 |  |  |  |
| 2. AE | 18 | 5788.96 | 2154 | 5824.96 | 3.06 | 4 | 0.55 |
| 3. CE | 16 | 5839.90 | 2156 | 5871.90 | 53.99 | 6 | 7.39E-10 |
| 4. E | 12 | 5918.22 | 2160 | 5942.22 | 132.32 | 10 | 1.57E-23 |
| *Synonyms (PGS, Sleep Disturbances, Age)* | | | | | | | |
| 1. ACE | 22 | 7541.05 | 2868 | 7585.05 |  |  |  |
| 2. AE | 18 | 7546.07 | 2872 | 7582.07 | 5.03 | 4 | 0.28 |
| 3. CE | 16 | 7555.62 | 2784 | 7587.62 | 14.58 | 6 | 0.02 |
| 4. E | 12 | 7714.66 | 2878 | 7738.66 | 173.62 | 10 | 4.95E-32 |
| *Symbol Digit (PGS, Sleep Duration, Age)* | | | | | | | |
| 1. ACE | 22 | 7194.86 | 2691 | 7238.86 |  |  |  |
| 2. AE | 18 | 7197.34 | 2695 | 7233.34 | 2.48 | 4 | 0.65 |
| 3. CE | 16 | 7229.29 | 2697 | 7261.29 | 34.42 | 6 | 5.57E-06 |
| 4. E | 12 | 7401.47 | 2701 | 7425.47 | 206.60 | 10 | 6.76E-39 |
| *Symbol Digit (PGS, Sleep Disturbances, Age)* | | | | | | | |
| 1. ACE | 23 | 10435.6 | 4029 | 10481.6 |  |  |  |
| 2. AE | 19 | 10440.9 | 4033 | 10478.9 | 5.33 | 4 | 0.25 |
| 3. CE | 17 | 10485.3 | 4035 | 10519.3 | 49.71 | 6 | 5.37E-09 |
| 4. E | 13 | 10747.7 | 4039 | 10773.7 | 312.08 | 10 | 4.34E-61 |

*Note.* Shaded rows indicate the best-fitting model based on AIC.

**Supplemental Table S6** Fit Statistics for Moderation Models (AD PGS without APOE)

| Models | K | 2LL | df | AIC | Δχ^2^ | *Δdf* | *p* |
| --- | --- | --- | --- | --- | --- | --- | --- |
| *Word List (PGS w/o APOE, Sleep Duration, Age)* | | | | | | | |
| 1. ACE | 23 | 13018.3 | 4706 | 13064.3 | NA | NA | NA |
| 2. AE | 19 | 13031.9 | 4710 | 13069.9 | 13.68 | 4 | 0.01 |
| 3. CE | 17 | 13028.3 | 4712 | 13062.3 | 10.04 | 6 | 0.12 |
| 4. E | 13 | 13103.3 | 4716 | 13129.3 | 85.04 | 10 | 5.12E-14 |
| *Word List (PGS w/o APOE, Sleep Disturbances, Age)* | | | | | | | |
| 1. ACE | 24 | 17962.8 | 6700 | 18010.8 | NA | NA | NA |
| 2. AE | 20 | 17965.7 | 6704 | 18005.7 | 2.85 | 4 | 0.58 |
| 3. CE | 18 | 17970.6 | 6706 | 18006.6 | 7.78 | 6 | 0.25 |
| 4. E | 14 | 18059.4 | 6710 | 18087.4 | 96.55 | 10 | 2.66E-16 |
| *Animal Naming (PGS w/o APOE, Sleep Duration, Age)* | | | | | | | |
| 1. ACE | 22 | 12339.6 | 4452 | 12383.6 | NA | NA | NA |
| 2. AE | 18 | 12342.1 | 4456 | 12378.1 | 2.57 | 4 | 0.63 |
| 3. CE | 16 | 12361 | 4458 | 12393.0 | 21.44 | 6 | 0.002 |
| 4. E | 12 | 12465.5 | 4462 | 12489.5 | 125.94 | 10 | 3.15E-22 |
| *Animal Naming (PGS w/o APOE, Sleep Disturbances, Age)* | | | | | | | |
| 1. ACE | 22 | 13449.0 | 4958 | 13493.0 | NA | NA | NA |
| 2. AE | 18 | 13452.3 | 4962 | 13488.3 | 3.32 | 4 | 0.51 |
| 3. CE | 16 | 13473.4 | 4964 | 13505.4 | 24.37 | 6 | 4.47E-04 |
| 4. E | 12 | 13595.7 | 4968 | 13619.7 | 146.67 | 10 | 1.80E-26 |
| *Digit Forward (PGS w/o APOE, Sleep Duration, Age)* | | | | | | | |
| 1. ACE | 23 | 13175.5 | 4781 | 13221.5 | NA | NA | NA |
| 2. AE | 19 | 13180.1 | 4785 | 13218.1 | 4.64 | 4 | 0.33 |
| 3. CE | 17 | 13199.0 | 4787 | 13233.0 | 23.50 | 6 | 6.45E-04 |
| 4. E | 13 | 13377.7 | 4791 | 13403.7 | 202.21 | 10 | 5.58E-38 |
| *Digit Forward (PGS w/o APOE, Sleep Disturbances, Age)* | | | | | | | |
| 1. ACE | 24 | 16870.2 | 6157 | 16918.2 | NA | NA | NA |
| 2. AE | 20 | 16879.5 | 6161 | 16919.5 | 9.24 | 4 | 0.06 |
| 3. CE | 18 | 16913.0 | 6163 | 16949.0 | 42.76 | 6 | 1.30E-07 |
| 4. E | 14 | 17145.2 | 6167 | 17173.2 | 274.97 | 10 | 2.99E-53 |
| *Digit Backward (PGS w/o APOE, Sleep Duration, Age)* | | | | | | | |
| 1. ACE | 23 | 13183.1 | 4778 | 13229.1 | NA | NA | NA |
| 2. AE | 19 | 13184.7 | 4782 | 13222.7 | 1.54 | 4 | 0.82 |
| 3. CE | 17 | 13197.2 | 4784 | 13231.2 | 14.09 | 6 | 0.03 |
| 4. E | 13 | 13325.3 | 4788 | 13351.3 | 142.21 | 10 | 1.48E-25 |
| *Digit Backward (PGS w/o APOE, Sleep Disturbances, Age)* | | | | | | | |
| 1. ACE | 24 | 13175.5 | 4781 | 13221.5 | NA | NA | NA |
| 2. AE | 20 | 13180.1 | 4785 | 13218.1 | 4.64 | 4 | 0.33 |
| 3. CE | 18 | 13199.0 | 4787 | 13233.0 | 23.50 | 6 | 6.45E-04 |
| 4. E | 14 | 13377.7 | 4791 | 13403.7 | 202.21 | 10 | 5.58E-38 |
| *Synonyms (PGS w/o APOE, Sleep Duration, Age)* | | | | | | | |
| 1. ACE | 22 | 5794.5 | 2150 | 5838.5 | NA N | A | NA |
| 2. AE | 18 | 5799.6 | 2154 | 5835.6 | 5.03 | 4 | 0.28 |
| 3. CE | 16 | 5842.6 | 2156 | 5874.6 | 48.08 | 6 | 1.14E-08 |
| 4. E | 12 | 5949.1 | 2160 | 5948.6 | 154.60 | 10 | 4.21202E-28 |
| *Synonyms (PGS w/o APOE, Sleep Disturbances, Age)* | | | | | | | |
| 1. ACE | 22 | 7534.4 | 2868 | 7578.4 | NA | NA | NA |
| 2. AE | 18 | 7540.7 | 2872 | 7576.7 | 6.26 | 4 | 0.18 |
| 3. CE | 16 | 7546.1 | 2874 | 7578.1 | 11.72 | 6 | 0.07 |
| 4. E | 12 | 7703.2 | 2878 | 7727.2 | 168.74 | 10 | 5.05E-31 |
| *Symbol Digit (PGS w/o APOE, Sleep Duration, Age)* | | | | | | | |
| 1. ACE | 22 | 7147.4 | 2691 | 7191.4 | NA | NA | NA |
| 2. AE | 18 | 7152.1 | 2695 | 7188.1 | 4.76 | 4 | 0.31 |
| 3. CE | 16 | 7179.7 | 2697 | 7211.7 | 32.35 | 6 | 1.40E-05 |
| 4. E | 12 | 7357.8 | 2701 | 7381.8 | 210.40 | 10 | 1.09E-39 |
| *Symbol Digit (PGS w/o APOE, Sleep Disturbances, Age)* | | | | | | | |
| 1. ACE | 23 | 10395.0 | 4029 | 10441.0 | NA | NA | NA |
| 2. AE | 19 | 10400.2 | 4033 | 10438.2 | 5.21 | 4 | 0.27 |
| 3. CE | 17 | 10445.4 | 4035 | 10479.4 | 50.39 | 6 | 3.92E-09 |
| 4. E | 13 | 10711.6 | 4039 | 10737.6 | 316.53 | 10 | 4.94E-62 |

*Note.* Shaded rows indicate the best-fitting model based on AIC.

**Supplemental Table S7** Parameter Estimates and 95% confidence intervals for AE Models: AD-PGS, Age, and Sleep Duration as moderators

| Cognitive Task | Est. | 95% CI | Est. | 95% CI | Est. | 95% CI |
| --- | --- | --- | --- | --- | --- | --- |
| ***Word List*** | A_p_ | | A_L_ | | E_0_ | |
|  | -0.02 | [-0.06, 0.02] | **0.49** | **[0.41, 0.55]** | **0.84** | **[0.80, 0.88]** |
| AD-PGS |  |  |  |  | B_Ap,E_ | |
|  | -- |  | -- |  | **-0.03** | **[-0.06, -0.01]** |
| Age | B_Age,Ap_ | | B_Age,AL_ | | BA_ge,E_ | |
|  | -0.04 | [-0.08, 0.01] | **0.13** | **[0.07, 0.21]** | **-0.05** | **[-0.08, -0.01]** |
| Sleep Duration | B_SD,Ap_ | | B_SD,AL_ | | B_SD,E_ | |
|  | -0.01 | [-0.05, 0.03] | 0.01 | [-0.08, 0.11] | 0.004 | [-0.05, 0.06] |
| ***Animal Naming*** | A_p_ | | A_L_ | | E_0_ | |
|  | -0.02 | [-0.07, 0.02] | **0.61** | **[0.56, 0.67]** | **0.78** | **[0.74, 0.82]** |
| AD-PGS |  |  |  |  | B_Ap,E_ | |
|  | -- |  | -- |  | -0.01 | [-0.04, 0.02] |
| Age | B_Age,Ap_ | | B_Age,AL_ | | BA_ge,E_ | |
|  | -0.003 | [-0.04, 0.04] | 0.03 | [-0.03, 0.09] | -0.01 | [-0.05, 0.02] |
| Sleep Duration | B_SD,Ap_ | | B_SD,AL_ | | B_SD,E_ | |
|  | -0.004 | [-0.04, 0.04] | -0.05 | [-0.12, 0.02] | 0.002 | [-0.05, 0.05] |
| ***Digit Forward*** | A_p_ | | A_L_ | | E_0_ | |
|  | 0.01 | [-0.03, 0.05] | **0.68** | **[0.63, 0.73]** | **0.72** | **[0.69, 0.76]** |
| AD-PGS |  |  |  |  | B_Ap,E_ | |
|  | -- |  | -- |  | -0.01 | [-0.04, 0.02] |
| Age | B_Age,Ap_ | | B_Age,AL_ | | BA_ge,E_ | |
|  | 0.02 | [-0.03, 0.07] | 0.05 | [-0.01, 0.10] | 0.01 | [-0.03, 0.05] |
| Sleep Duration | B_SD,Ap_ | | B_SD,AL_ | | B_SD,E_ | |
|  | -0.03 | [-0.09, 0.02] | -0.03 | [-0.09, 0.02] | 0.003 | [-0.04, 0.05] |
| ***Digit Backward*** | A_p_ | | A_L_ | | E_0_ | |
|  | 0.02 | [-0.02, 0.06] | **0.62** | **[0.56, 0.67]** | **0.76** | **[0.73, 0.80]** |
| AD-PGS |  |  |  |  | B_Ap,E_ | |
|  | -- |  | -- |  | **-0.04** | **[-0.06, -0.01]** |
| Age | B_Age,Ap_ | | B_Age,AL_ | | BA_ge,E_ | |
|  | 0.02 | [-0.02, 0.06] | **-0.07** | **[-0.14, -0.01]** | 0.03 | [-0.01, 0.07] |
| Sleep Duration | B_SD,Ap_ | | B_SD,AL_ | | B_SD,E_ |  |
|  | -0.01 | [-0.04, 0.03] | 0.01 | [-0.05, 0.07] | **-0.04** | **[-0.09, -0.001]** |
| ***Synonyms*** | A_p_ | | A_L_ | | E_0_ | |
|  | 0.02 | [-0.04, 0.08] | **0.69** | **[0.62, 0.75]** | **0.66** | **[0.62, 0.71]** |
| AD-PGS |  |  |  |  | B_Ap,E_ | |
|  | -- |  | -- |  | **-0.07** | **[-0.11, -0.02]** |
| Age | B_Age,Ap_ | | B_Age,AL_ | | BA_ge,E_ | |
|  | 0.00 | [-0.10, 0.10] | **0.25** | **[0.17, 0.34]** | **0.06** | **[0.01, 0.12]** |
| Sleep Duration | B_SD,Ap_ | | B_SD,AL_ | | B_SD,E_ | |
|  | -0.004 | [-0.05, 0.03] | -0.006 | [-0.07, 0.07] | 0.01 | [-0.04, 0.06] |
| ***Symbol Digit*** | A_p_ | | A_L_ | | E_0_ | |
|  | -0.04 | [-0.09, 0.02] | **0.75** | **[0.69, 0.80]** | **0.56** | **[0.52, 0.60]** |
| AD-PGS |  |  |  |  | B_Ap,E_ | |
|  | -- |  | -- |  | 0.02 | [-0.02, 0.06] |
| Age | B_Age,Ap_ | | B_Age,AL_ | | BA_ge,E_ | |
|  | 0.00 | [-0.06, 0.06] | **0.10** | **[0.05, 0.16]** | 0.04 | [-0.003, 0.09] |
| Sleep Duration | B_SD,Ap_ | | B_SD,AL_ | | B_SD,E_ | |
|  | -0.004 | [-0.05, 0.05] | -0.05 | [-0.10, 0.01] | 0.04 | [-0.01, 0.09] |

Note. A=additive genetic, E=nonshared environment, PGS-AD-Polygenic Score, Est.=Estimate. Moderation parameter terms include the AD-PGS, sleep, and age.

**Supplemental Table S8** Parameter Estimates and 95% confidence intervals for ACE Models: AD-PGS, Age, and Sleep Duration as moderators

| Cognitive Task | Est. | 95% CI | Est. | 95% CI | Est. | | 95% CI | Est. | 95% CI |
| --- | --- | --- | --- | --- | --- | --- | --- | --- | --- |
| ***Word List*** | A_p_ | | A_L_ | | C_0_ | | | E_0_ | |
|  | -0.02 | [-0.06, 0.02] | 0.23 | [-0.27, 0.50] | 0.36 | [-0.05, 0.47] | | **0.85** | **[0.80, 0.89]** |
| AD-PGS |  |  |  |  | B_Ap,C_ | | | B_Ap,E_ | |
|  | -- |  | -- |  | 0.03 | | [-0.06, 0.17] | **-0.04** | **[-0.08, 0]** |
| Age | B_Age,Ap_ | | B_Age,AL_ | | BA_ge,C_ | | | BA_ge,E_ | |
|  | -0.04 | [-0.08, 0.01] | -0.08 | [-0.24, 0.17] | **0.14** | | **[0.03, 0.24]** | -0.02 | [-0.07, 0.02] |
| Sleep Duration | B_SD,Ap_ | | B_SD,AL_ | | B_SD,C_ | | | B_SD,E_ | |
|  | -0.01 | [-0.05, 0.03] | -0.13 | [-0.25, 0.25] | 0.06 | | [-0.07, 0.22] | 0.02 | [-0.04, 0.07] |
| ***Animal Naming*** | A_p_ | | A_L_ | | C_0_ | | | E_0_ | |
|  | -0.02 | [-0.06, 0.02] | **0.55** | **[0.38, 0.65]** | 0.23 | | [-0.42, 0.42] | **0.78** | **[0.74, 0.82]** |
| AD-PGS |  |  |  |  | B_Ap,C_ | | | B_Ap,E_ | |
|  | -- |  | -- |  | -0.04 | | [-0.2, 0.2] | -0.01 | [-0.04, 0.03] |
| Age | B_Age,Ap_ | | B_Age,AL_ | | BA_ge,C_ | | | BA_ge,E_ | |
|  | -0.001 | [-0.04, 0.04] | 0.08 | [-0.02, 0.17] | -0.10 | | [-0.21, 0.21] | -0.02 | [-0.05, 0.02] |
| Sleep Duration | B_SD,Ap_ | | B_SD,AL_ | | B_SD,C_ | | | B_SD,E_ | |
|  | -0.01 | [-0.05, 0.03] | -0.08 | [-0.17, 0.06] | 0.05 | | [-0.19, 0.18] | 0.004 | [-0.05, 0.05] |
| ***Digit Forward*** | A_p_ | | A_L_ | | C_0_ | | | E_0_ | |
|  | 0.01 | [-0.03, 0.05] | **0.63** | **[0.56, 0.69]** | -0.11 | | [-0.26, 0.08] | **0.72** | **[0.68, 0.76]** |
| AD-PGS |  |  |  |  | B_Ap,C_ | | | B_Ap,E_ | |
|  | -- |  | -- |  | **0.200** | | **[0.09, 0.28]** | -0.01 | [-0.04, 0.03] |
| Age | B_Age,Ap_ | | B_Age,AL_ | | BA_ge,C_ | | | BA_ge,E_ | |
|  | 0.02 | [-0.03, 0.07] | 0.04 | [-0.03, 0.1] | -0.03 | | [-0.13, 0.09] | 0.01 | [-0.02, 0.05] |
| Sleep Duration | B_SD,Ap_ | | B_SD,AL_ | | B_SD,C_ | | | B_SD,E_ | |
|  | -0.04 | [-0.08, 0.004] | -0.05 | [-0.12, 0.01] | **-0.11** | | **[-0.19, -0.01]** | 0.007 | [-0.04, 0.05] |
| ***Digit Backward*** | A_p_ | | A_L_ | | C_0_ | | | E_0_ | |
|  | 0.02 | [-0.02, 0.06] | **0.54** | **[0.36, 0.65]** | 0.25 | | [-0.44, 0.44] | **0.77** | **[0.73, 0.81]** |
| AD-PGS |  |  |  |  | B_Ap,C_ | | | B_Ap,E_ | |
|  | -- |  | -- |  | 0.06 | | [-0.2, 0.2] | **-0.05** | **[-0.08, -0.01]** |
| Age | B_Age,Ap_ | | B_Age,AL_ | | B_Age,C_ | | | BA_ge,E_ | |
|  | 0.02 | [-0.02, 0.07] | -0.04 | [-0.12, 0.04] | -0.08 | | [-0.16, 0.16] | 0.03 | [-0.01, 0.06] |
| Sleep Duration | B_SD,Ap_ | | B_SD,AL_ | | B_SD,C_ | | | B_SD,E_ |  |
|  | -0.01 | [-0.05, 0.03] | -0.02 | [-0.1, 0.06] | 0.05 | | [-0.06, 0.14] | **-0.04** | **[-0.09, -0.001]** |
|  | A_p_ | | A_L_ | | C_0_ | | | E_0_ | |
|  | 0.02 | [-0.05, 0.08] | **0.62** | **[0.45, 0.72]** | -0.08 | | [-0.38, 0.38] | **0.67** | **[0.62, 0.72]** |
| AD-PGS |  |  |  |  | B_Ap,C_ | | | B_Ap,E_ | |
|  | -- |  | -- |  | -0.01 | | [-0.12, 0.12] | **-0.07** | **[-0.11, -0.02]** |
| Age | B_Age,Ap_ | | B_Age,AL_ | | B_Age,C_ | | | BA_ge,E_ | |
|  | -0.01 | [-0.06, 0.04] | 0.01 | [-0.09, 0.13] | 0.05 | | [-0.16, 0.19] | 0.01 | [-0.05, 0.06] |
| Sleep Duration | B_SD,Ap_ | | B_SD,AL_ | | B_SD,C_ | | | B_SD,E_ | |
|  | -0.005 | [-0.06, 0.05] | -0.04 | [-0.11, 0.03] | 0.03 | | [-0.16, 0.16] | 0.04 | [-0.01, 0.09] |
| ***Symbol Digit*** | A_p_ | | A_L_ | | C_0_ | | | E_0_ | |
|  | -0.04 | [-0.09, 0.02] | **0.72** | **[0.58, 0.78]** | -0.01 | | [-0.42, 0.42] | **0.56** | **[0.52, 0.61]** |
| AD-PGS |  |  |  |  | B_Ap,C_ | | | B_Ap,E_ | |
|  | -- |  | -- |  | 0.07 | | [-0.22, 0.22] | 0.02 | [-0.02, 0.06] |
| Age | B_Age,Ap_ | | B_Age,AL_ | | B_Age,C_ | | | BA_ge,E_ | |
|  | 0.00 | [-0.06, 0.07] | 0.10 | [-0.02, 0.22] | 0.20 | | [-0.35, 0.35] | 0.04 | [-0.01, 0.09] |
| Sleep Duration | B_SD,Ap_ | | B_SD,AL_ | | B_SD,C_ | | | B_SD,E_ | |
|  | -0.005 | [-0.06, 0.05] | -0.04 | [-0.11, 0.03] | 0.03 | | [-0.16, 0.16] | 0.04 | [-0.01, 0.09] |

*Note.* A=additive genetic, C=shared environment, E=nonshared environment, PGS-AD-Polygenic Score. Moderation parameter terms include the AD-PGS, sleep, and age.

**Supplemental Table S9** Parameter Estimates and 95% Confidence Intervals for AE Models: AD-PGS (without SNPs in the *APOE* region), Age, and Sleep Duration as moderators

| Cognitive Task | Est. | 95% CI | Est. | 95% CI | Est. | 95% CI |
| --- | --- | --- | --- | --- | --- | --- |
| ***Word List*** | A_p_ | | A_L_ | | E_0_ | |
|  | -0.01 | [-0.05, 0.03] | **0.49** | **[0.41, 0.55]** | **0.84** | **[0.80, 0.88]** |
| AD-PGS |  |  |  |  | B_Ap,E_ | |
|  | -- | -- | -- | -- | -0.01 | [-0.04, 0.01] |
| Age | B_Age,Ap_ | | B_Age,AL_ | | BA_ge,E_ | |
|  | 0.02 | [-0.02, 0.07] | **0.13** | **[0.07, 0.21]** | **-0.04** | **[-0.08, 0]** |
| Sleep Dur. | B_SD,Ap_ | | B_SD,AL_ | | B_SD,E_ | |
|  | 0.02 | [-0.02, 0.06] | 0.01 | [-0.08, 0.11] | 0.01 | [-0.05, 0.06] |
| ***Animal Naming*** | A_p_ | | A_L_ | | E_0_ | |
|  | -0.02 | [-0.06, 0.02] | **0.62** | **[0.56, 0.67]** | **0.78** | **[0.74, 0.82]** |
| AD-PGS |  |  |  |  | B_Ap,E_ | |
|  | -- | -- | -- | -- | 0.01 | [-0.02, 0.04] |
| Age | B_Age,Ap_ | | B_Age,AL_ | | BA_ge,E_ | |
|  | -0.02 | [-0.07, 0.02] | 0.03 | [-0.03, 0.08] | -0.01 | [-0.05, 0.02] |
| Sleep Dur. | B_SD,Ap_ | | B_SD,AL_ | | B_SD,E_ | |
|  | -0.03 | [-0.07, 0.01] | -0.04 | [-0.12, 0.03] | -0.01 | [-0.06, 0.04] |
| ***Digit Forward*** | A_p_ | | A_L_ | | E_0_ | |
|  | -0.03 | [-0.07, 0.01] | **0.67** | **[0.62, 0.72]** | **0.73** | **[0.69, 0.76]** |
| AD-PGS |  |  |  |  | B_Ap,E_ | |
|  | -- | -- | -- | -- | -0.04 | [-0.06, 0.01] |
| Age | B_Age,Ap_ | | B_Age,AL_ | | BA_ge,E_ | |
|  | 0.00 | [-0.04, 0.04] | 0.04 | [-0.01, 0.01] | 0.01 | [-0.03, 0.05] |
| Sleep Dur. | B_SD,Ap_ | | B_SD,AL_ | | B_SD,E_ | |
|  | -0.02 | [-0.06, 0.02] | -0.04 | [-0.10, 0.02] | 0.01 | [-0.04, 0.05] |
| ***Digit Backward*** | A_p_ | | A_L_ | | E_0_ | |
|  | 0.005 | [-0.04, 0.05] | **0.61** | **[0.56, 0.67]** | **0.76** | **[0.73, 0.80]** |
| AD-PGS |  |  |  |  | B_Ap,E_ | |
|  | -- | -- | -- | -- | -0.02 | [-0.05, 0.01] |
| Age | B_Age,Ap_ | | B_Age,AL_ | | BA_ge,E_ | |
|  | 0.02 | [-0.02, 0.06] | **-0.06** | **[-0.13, 0]** | 0.03 | [-0.01, 0.07] |
| Sleep Dur. | B_SD,Ap_ |  | B_SD,AL_ |  | B_SD,E_ |  |
|  | -0.02 | [-0.06, 0.02] | 0.002 | [-0.06, 0.06] | -0.04 | [-0.08, 0.002] |
| ***Synonyms*** | A_p_ | | A_L_ | | E_0_ | |
|  | -0.02 | [-0.08, 0.04] | **0.68** | **[0.61, 0.75]** | **0.67** | **[0.62, 0.72]** |
| AD-PGS |  |  |  |  | B_Ap,E_ | |
|  | -- | -- | -- | -- | 0.01 | [-0.03, 0.05] |
| Age | B_Age,Ap_ | | B_Age,AL_ | | BA_ge,E_ | |
|  | -0.01 | [-0.10, 0.09] | **0.25** | **[0.17, 0.34]** | **0.06** | **[0.01, 0.12]** |
| Sleep Dur. | B_SD,Ap_ | | B_SD,AL_ | | B_SD,E_ | |
|  | 0.02 | [-0.03, 0.08] | 0.02 | [-0.05, 0.09] | -0.01 | [-0.06, 0.04] |
| ***Symbol Digit*** | A_p_ | | A_L_ | | E_0_ | |
|  | -0.01 | [-0.06, 0.04] | **0.74** | **[0.69, 0.80]** | **0.56** | **[0.52, 0.80]** |
| AD-PGS |  |  |  |  | B_Ap,E_ | |
|  | -- | -- | -- | -- | -0.04 | [-0.07, 0.001] |
| Age | B_Age,Ap_ | | B_Age,AL_ | | BA_ge,E_ | |
|  | 0.03 | [-0.03, 0.09] | **0.10** | **[0.04, 0.16]** | 0.04 | [-0.002, 0.09] |
| Sleep Dur. | B_SD,Ap_ | | B_SD,AL_ | | B_SD,E_ | |
|  | 0.005 | [-0.04, 0.05] | -0.04 | [-0.10, 0.01] | 0.04 | [-0.01, 0.09] |

*Note.* A_p_ = genetic influences attributable to the AD-PGS, A_L_= additive genetic influences, E= non-shared environmental influences, Est.=Estimate, Sleep Dur.=Sleep Duration. Moderation parameter terms include the AD-PGS, age, and sleep duration. Bolded parameters indicate *p <* .05.

**Supplemental Table S10** Parameter Estimates and 95% confidence intervals for ACE Models: AD-PGS (excluding SNPs within the *APOE* region), Age, and Sleep Duration as moderators

| Cognitive Task | Est. | 95% CI | Est. | 95% CI | Est. | | 95% CI | Est. | 95% CI |
| --- | --- | --- | --- | --- | --- | --- | --- | --- | --- |
| ***Word List*** | A_p_ | | A_L_ | | C_0_ | | | E_0_ | |
|  | -0.01 | [-0.05, 0.03] | 0.14 | [-0.23, 0.44] | **0.40** | **[0.18, 0.47]** | | **0.85** | **[0.81, 0.89]** |
| AD-PGS |  |  |  |  | B_Ap,C_ | | | B_Ap,E_ | |
|  | -- |  | -- |  | -0.06 | | [-0.14, 0] | 0 | [-0.03, 0.03] |
| Age | B_Age,Ap_ | | B_Age,AL_ | | BA_ge,C_ | | | BA_ge,E_ | |
|  | 0.02 | [-0.02, 0.07] | -0.12 | [-0.24, 0.12] | 0.12 | | [0.02, 0.21] | -0.03 | [-0.07, 0.02] |
| Sleep Dur. | B_SD,Ap_ | | B_SD,AL_ | | B_SD,C_ | | | B_SD,E_ | |
|  | 0.02 | [-0.03, 0.06] | **-0.15** | **[-0.26, -0.01]** | 0.04 | | [-0.08, 0.17] | 0.02 | [-0.03, 0.07] |
| ***Animal Naming*** | A_p_ | | A_L_ | | C_0_ | | | E_0_ | |
|  | -0.02 | [-0.06, 0.022] | **0.55** | **[0.38, 0.65]** | 0.22 | | [-0.41, 0.41] | **0.78** | **[0.74, 0.82]** |
| AD-PGS |  |  |  |  | B_Ap,C_ | | | B_Ap,E_ | |
|  | -- |  | -- |  | 0.04 | | [-0.17, 0.17] | 0.002 | [-0.03,0.03] |
| Age | B_Age,Ap_ | | B_Age,AL_ | | BA_ge,C_ | | | BA_ge,E_ | |
|  | -0.02 | [-0.07, 0.02] | 0.07 | [-0.02, 0.17] | -0.10 | | [-0.21, 0.21] | -0.01 | [-0.05, 0.02] |
| Sleep Dur. | B_SD,Ap_ | | B_SD,AL_ | | B_SD,C_ | | | B_SD,E_ | |
|  | -0.03 | [-0.07, 0.01] | -0.08 | [-0.17, 0.04] | 0.06 | | [-0.18, 0.18] | -0.003 | [-0.05, 0.04] |
| ***Digit Forward*** | A_p_ | | A_L_ | | C_0_ | | | E_0_ | |
|  | -0.03 | [-0.07, 0.01] | **0.63** | **[0.47, 0.70]** | 0.15 | | [-0.04, 0.44] | **0.73** | **[0.69, 0.77]** |
| AD-PGS |  |  |  |  | B_Ap,C_ | | | B_Ap,E_ | |
|  | -- |  | -- |  | -0.17 | | [-0.28, 0.02] | -0.02 | [-0.05, 0.01] |
| Age | B_Age,Ap_ | | B_Age,AL_ | | BA_ge,C_ | | | BA_ge,E_ | |
|  | 0 | [-0.04, 0.05] | 0.06 | [-0.01, 0.14] | -0.04 | | [-0.17, 0.10] | 0.01 | [-0.03, 0.05] |
| Sleep Dur. | B_SD,Ap_ | | B_SD,AL_ | | B_SD,C_ | | | B_SD,E_ | |
|  | -0.02 | [-0.06, 0.02] | -0.04 | [-0.12, 0.04] | 0.03 | | [-0.11, 0.14] | 0.004 | [-0.04, 0.05] |
| ***Digit Backward*** | A_p_ | | A_L_ | | C_0_ | | | E_0_ | |
|  | 0.01 | [-0.04, 0.05] | **0.54** | **[0.36, 0.66]** | 0.26 | | [-0.44, 0.44] | **0.77** | **[0.73, 0.81]** |
| AD-PGS |  |  |  |  | B_Ap,C_ | | | B_Ap,E_ | |
|  | -- |  | -- |  | -0.04 | | [-0.20, 0.20] | -0.01 | [-0.05, 0.02] |
| Age | B_Age,Ap_ | | B_Age,AL_ | | B_Age,C_ | | | BA_ge,E_ | |
|  | 0.02 | [-0.02, 0.06] | -0.04 | [-0.13, 0.04] | -0.07 | | [-0.15, 0.15] | 0.03 | [-0.01, 0.07] |
| Sleep Dur. | B_SD,Ap_ | | B_SD,AL_ | | B_SD,C_ | | | B_SD,E_ |  |
|  | -0.02 | [-0.06, 0.02] | -0.01 | [-0.10, 0.07] | 0.04 | | [-0.14, 0.14] | -0.04 | [-0.08, 0] |
| ***Synonyms*** | A_p_ | | A_L_ | | C_0_ | | | E_0_ | |
|  | -0.02 | [-0.08, 0.04] | 0.57 | [-0.22, 0.69] | -0.16 | | [-0.57, 0.13] | **0.68** | **[0.63, 0.73]** |
| AD-PGS |  |  |  |  | B_Ap,C_ | | | B_Ap,E_ | |
|  | -- |  | -- |  | -0.02 | | [-0.14, 0.12] | 0.02 | [-0.03, 0.07] |
| Age | B_Age,Ap_ | | B_Age,AL_ | | B_Age,C_ | | | BA_ge,E_ | |
|  | 0 | [-0.10, 0.10] | **0.28** | **[0.09, 0.71]** | **0.31** | | **[0.02, 0.51]** | **0.06** | **[0.01, 0.13]** |
| Sleep Dur. | B_SD,Ap_ | | B_SD,AL_ | |  | | B_SD,C_ | B_SD,E_ |  |
|  | 0.02 | [-0.03, 0.08] | 0.05 | [-0.07, 0.18] | 0.05 | | [-0.11, 0.18] | -0.01 | [-0.06, 0.04] |
| ***Symbol Digit*** | A_p_ | | A_L_ | | C_0_ | | | E_0_ | |
|  | -0.01 | [-0.07, 0.04] | **0.69** | **[0.59, 0.77]** | 0.01 | | [-0.22, 0.39] | **0.57** | **[0.53, 0.62]** |
| AD-PGS |  |  |  |  | B_Ap,C_ | | | B_Ap,E_ | |
|  | -- |  | -- |  | 0.13 | | [-0.04, 0.26] | -0.04 | [-0.08, 0] |
| Age | B_Age,Ap_ | | B_Age,AL_ | | B_Age,C_ | | | BA_ge,E_ | |
|  | 0.02 | [-0.04, 0.08] | 0.10 | [0, 0.22] | -0.22 | | [-0.36, 0.06] | 0.04 | [-0.003, 0.09] |
| Sleep Dur. | B_SD,Ap_ | | B_SD,AL_ | | B_SD,C_ | | | B_SD,E_ | |
|  | 0 | [-0.05, 0.05] | -0.05 | [-0.11, 0.02] | -0.03 | | [-0.13, 0.08] | 0.04 | [-0.01, 0.09] |

*Note.* A=additive genetic, C=shared environment, E=nonshared environment, PGS-AD-Polygenic Score. Sleep Dur.=Sleep Duration. Moderation parameter terms include the AD-PGS, sleep duration, and age.

**Supplemental Table S11** Parameter Estimates and 95% confidence intervals for ACE Models: AD-PGS, Age, and Sleep Disturbances as moderators

| Cognitive Task | Est. | 95% CI | Est. | 95% CI | Est. | | 95% CI | Est. | 95% CI |
| --- | --- | --- | --- | --- | --- | --- | --- | --- | --- |
| ***Word List*** | A_p_ | | A_L_ | | C_0_ | | | E_0_ | |
|  | **-0.04** | **[-0.07, -0.01]** | **0.37** | **[0.09, 0.51]** | 0.26 | [-0.4, 0.4] | | **0.77** | **[0.74, 0.81]** |
| AD-PGS |  |  |  |  | B_Ap,C_ | | | B_Ap,E_ | |
|  | -- |  | -- |  | 0.02 | | [-0.15, 0.15] | -0.03 | [-0.06, 0.001] |
| Age | B_Age,Ap_ | | B_Age,AL_ | | BA_ge,C_ | | | BA_ge,E_ | |
|  | **-0.04** | **[-0.07, -0.01]** | 0.06 | [-0.08, 0.18] | 0.06 | | [-0.21, 0.21] | -0.01 | [-0.04, 0.02] |
| Sleep Dist. | B_SD,Ap_ | | B_SD,AL_ | | B_SD,C_ | | | B_SD,E_ | |
|  | -0.005 | [-0.04, 0.03] | 0.10 | [-0.13, 0.19] | -0.06 | | [-0.15, 0.15] | -0.03 | [-0.07, 0.007] |
| ***Animal Naming*** | A_p_ | | A_L_ | | C_0_ | | | E_0_ | |
|  | -0.03 | [-0.07, 0.01] | **0.56** | **[0.48, 0.62]** | -0.12 | | [-0.29, 0.15] | **0.73** | **[0.69, 0.76]** |
| AD-PGS |  |  |  |  | B_Ap,C_ | | | B_Ap,E_ | |
|  | -- |  | -- |  | **0.13** | | **[0.01, 0.22]** | -0.01 | [-0.04, 0.02] |
| Age | B_Age,Ap_ | | B_Age,AL_ | | BA_ge,C_ | | | BA_ge,E_ | |
|  | -0.01 | [-0.05, 0.03] | -0.01 | [-0.04, 0.06] | 0.02 | | [-0.1, 0.12] | **-0.04** | **[-0.07, -0.001]** |
| Sleep Dist. | B_SD,Ap_ | | B_SD,AL_ | | B_SD,C_ | | | B_SD,E_ | |
|  | 0.02 | [-0.01, 0.06] | 0.03 | [-0.04, 0.11] | 0.11 | | [-0.03, 0.19] | 0.03 | [-0.02, 0.07] |
| ***Digit Forward*** | A_p_ | | A_L_ | | C_0_ | | | E_0_ | |
|  | 0.003 | [-0.03, 0.04] | **0.64** | **[0.49, 0.69]** | -0.10 | | [-0.39, 0.34] | **0.70** | **[0.67, 0.74]** |
| AD-PGS |  |  |  |  | B_Ap,C_ | | | B_Ap,E_ | |
|  | -- |  | -- |  | 0.10 | | [-0.04, 0.23] | -0.01 | [-0.04, 0.02] |
| Age | B_Age,Ap_ | | B_Age,AL_ | | BA_ge,C_ | | | BA_ge,E_ | |
|  | -0.01 | [-0.05, 0.03] | -0.02 | [-0.07, 0.05] | -0.03 | | [-0.14, 0.08] | -0.01 | [-0.04, 0.02] |
| Sleep Dist. | B_SD,Ap_ | | B_SD,AL_ | | B_SD,C_ | | | B_SD,E_ | |
|  | -0.02 | [-0.05, 0.02] | 0.07 | [-0.09, 0.18] | **0.16** | | **[0.06, 0.24]** | -0.02 | [-0.06, 0.02] |
|  |  | |  | |  | | |  | |
| ***Digit Backward*** | A_p_ | | A_L_ | | C_0_ | | | E_0_ | |
|  | 0.02 | [-0.02, 0.05] | **0.58** | **[0.49, 0.63]** | 0.12 | | [-0.05, 0.3] | **0.71** | **[0.68, 0.75]** |
| AD-PGS |  |  |  |  | B_Ap,C_ | | | B_Ap,E_ | |
|  | -- |  | -- |  | **0.11** | | **[0.04, 0.19]** | **-0.03** | **[-0.06, -0.004]** |
| Age | B_Age,Ap_ | | B_Age,AL_ | | B_Age,C_ | | | BA_ge,E_ | |
|  | -0.01 | [-0.04, 0.03] | -0.05 | [-0.1, 0.01] | -0.05 | | [-0.14, 0.03] | -0.02 | [-0.05, 0.01] |
| Sleep Dist. | B_SD,Ap_ | | B_SD,AL_ | | B_SD,C_ | | | B_SD,E_ |  |
|  | 0.02 | [-0.01, 0.06] | -0.03 | [-0.14, 0.06] | **0.24** | | **[0.17, 0.3]** | -0.02 | [-0.06, 0.02] |
| ***Synonyms*** | A_p_ | | A_L_ | | C_0_ | | | E_0_ | |
|  | -0.02 | [-0.07, 0.03] | **0.56** | **[0.31, 0.69]** | **0.38** | | **[0.02, 0.57]** | **0.64** | **[0.59, 0.69]** |
| AD-PGS |  |  |  |  | B_Ap,C_ | | | B_Ap,E_ | |
|  | -- |  | -- |  | 0.01 | | [-0.06, 0.1] | **-0.05** | **[-0.09, -0.01]** |
| Age | B_Age,Ap_ | | B_Age,AL_ | | B_Age,C_ | | | BA_ge,E_ | |
|  | -0.05 | [-0.11, 0.01] | **0.26** | **[0.12, 0.41]** | 0.22 | | [-0.01, 0.35] | **0.11** | **[0.07, 0.17]** |
| Sleep Dist. | B_SD,Ap_ | | B_SD,AL_ | | B_SD,C_ | | | B_SD,E_ | |
|  | -0.02 | [-0.06, 0.02] | 0.07 | [-0.16, 0.15] | -0.07 | | [-0.18, 0.17] | 0.02 | [-0.02, 0.08] |
| ***Symbol Digit*** | A_p_ | | A_L_ | | C_0_ | | | E_0_ | |
|  | -0.03 | [-0.07, 0.01] | **0.66** | **[0.56, 0.73]** | 0.23 | | [-0.39, 0.40] | **0.52** | **[0.48, 0.56]** |
| AD-PGS |  |  |  |  | B_Ap,C_ | | | B_Ap,E_ | |
|  | -- |  | -- |  | 0.06 | | [-0.14, 0.17] | -0.001 | [-0.03, 0.03] |
| Age | B_Age,Ap_ | | B_Age,AL_ | | B_Age,C_ | | | BA_ge,E_ | |
|  | 0.005 | [-0.04, 0.05] | **0.09** | **[0.02, 0.16]** | -0.03 | | [-0.21, 0.21] | 0.02 | [-0.02, 0.06] |
| Sleep Dist. | B_SD,Ap_ | | B_SD,AL_ | | B_SD,C_ | | | B_SD,E_ | |
|  | 0.02 | [-0.02, 0.06] | 0.02 | [-0.14, 0.09] | -0.12 | | [-0.21, 0.22] | 0.01 | [-0.03, 0.06] |

*Note.* A=additive genetic, C=shared environment, E=nonshared environment, PGS-AD-Polygenic Score. Sleep Dist.=Sleep Disturbance. Moderation parameter terms include the AD-PGS, sleep, and age.

**Supplemental Table S12** Parameter Estimates and 95% Confidence Intervals for AE Models: AD-PGS (excluding SNPs within the *APOE* region), Age, and Sleep Disturbances as moderators

| Cognitive Task | Est. | 95% CI | Est. | 95% CI | Est. | 95% CI |
| --- | --- | --- | --- | --- | --- | --- |
| ***Word List*** | A_p_ | | A_L_ | | E_0_ | |
|  | -0.003 | [-0.03, 0.03] | **0.48** | **[0.42, 0.53]** | **0.77** | **[0.74, 0.80]** |
| AD-PGS |  |  |  |  | B_Ap,E_ | |
|  | -- | -- | -- | **--** | 0.004 | [-0.02, 0.03] |
| Age | B_Age,Ap_ | | B_Age,AL_ | | BA_ge,E_ | |
|  | 0.02 | [-0.02, 0.05] | **0.09** | **[0.04, 0.14]** | -0.01 | [-0.04, 0.02] |
| Sleep Dist. | B_SD,Ap_ | | B_SD,AL_ | | B_SD,E_ | |
|  | -0.005 | [-0.03, 0.02] | 0.05 | [-0.02, 0.11] | -0.03 | [-0.07, 0.01] |
| ***Animal Naming*** | A_p_ | | A_L_ | | E_0_ | |
|  | -0.02 | [-0.05, 0.02] | **0.60** | **[0.54, 0.65]** | **0.73** | **[0.70, 0.77]** |
| AD-PGS |  |  |  |  | B_Ap,E_ | |
|  | -- | -- | -- | -- | 0.01 | [-0.01, 0.04] |
| Age | B_Age,Ap_ | | B_Age,AL_ | | BA_ge,E_ | |
|  | -0.003 | [-0.04, 0.04] | 0.01 | [-0.04, 0.06] | **-0.04** | **[-0.07, -0.01]** |
| Sleep Dist. | B_SD,Ap_ | | B_SD,AL_ | | B_SD,E_ | |
|  | 0.03 | [-0.01, 0.07] | -0.004 | [-0.07, 0.06] | 0.03 | [-0.01, 0.08] |
| ***Digit Forward*** | A_p_ | | A_L_ | | E_0_ | |
|  | **-0.04** | **[-0.07, -0.002]** | **0.66** | **[0.62, 0.71]** | **0.71** | **[0.69, 0.75]** |
| AD-PGS |  |  |  |  | B_Ap,E_ | |
|  | -- | -- | -- | -- | **-0.004** | **[-0.06, -0.01]** |
| Age | B_Age,Ap_ | | B_Age,AL_ | | BA_ge,E_ | |
|  | -0.01 | [-0.05, 0.02] | -0.01 | [-0.05, 0.03] | -0.01 | [-0.04, 0.02] |
| Sleep Dist. | B_SD,Ap_ | | B_SD,AL_ | | B_SD,E_ | |
|  | -0.03 | [-0.06, 0.01] | 0.05 | [-0.01, 0.10] | -0.03 | [-0.07, 0.01] |
| ***Digit Backward*** | A_p_ | | A_L_ | | E_0_ | |
|  | -0.01 | [-0.05, 0.02] | **0.61** | **[0.56, 0.66]** | **0.74** | **[0.71, 0.77]** |
| AD-PGS |  |  |  |  | B_Ap,E_ | |
|  | -- | -- | -- | **--** | -0.02 | [-0.045, 0.004] |
| Age | B_Age,Ap_ | | B_Age,AL_ | | BA_ge,E_ | |
|  | -0.01 | [-0.05, 0.02] | **-0.06** | **[-0.11, -0.01]** | -0.03 | [-0.06, 0.01] |
| Sleep Dist. | B_SD,Ap_ |  | B_SD,AL_ |  | B_SD,E_ |  |
|  | 0.02 | [-0.01, 0.05] | 0.03 | [-0.04, 0.10] | -0.03 | [-0.08, 0.02] |
| ***Synonyms*** | A_p_ | | A_L_ | | E_0_ | |
|  | -0.03 | [-0.08, 0.02] | **0.68** | **[0.63, 0.74]** | **0.63** | **[0.59, 0.67]** |
| AD-PGS |  |  |  |  | B_Ap,E_ | |
|  | -- | -- | -- | **--** | -0.01 | [-0.04, 0.03] |
| Age | B_Age,Ap_ | | B_Age,AL_ | | BA_ge,E_ | |
|  | -0.01 | [-0.07, 0.05] | **0.33** | **[0.27, 0.40]** | **0.10** | **[0.05, 0.15]** |
| Sleep Dist. | B_SD,Ap_ | | B_SD,AL_ | | B_SD,E_ | |
|  | -0.03 | [-0.07, 0.01] | 0.02 | [-0.04, 0.07] | 0.03 | [-0.01, 0.07] |
| ***Symbol Digit*** | A_p_ | | A_L_ | | E_0_ | |
|  | -0.04 | [-0.08, 0.004] | **0.70** | **[0.66, 0.74]** | **0.52** | **[0.49, 0.55]** |
| AD-PGS |  |  |  |  | B_Ap,E_ | |
|  | -- | -- | -- | -- | -0.02 | [-0.05, 0.01] |
| Age | B_Age,Ap_ | | B_Age,AL_ | | BA_ge,E_ | |
|  | 0.003 | [-0.04, 0.04] | **0.08** | **[0.04, 0.12]** | 0.02 | [-0.02, 0.05] |
| Sleep Dist. | B_SD,Ap_ | | B_SD,AL_ | | B_SD,E_ | |
|  | -0.003 | [-0.04, 0.03] | -0.03 | [-0.07, 0.02] | 0.02 | [-0.02, 0.07] |

*Note.* A_p_ = genetic influences attributable to the AD-PGS, A_L_= additive genetic influences, E= non-shared environmental influences, Est.=Estimate. Moderation parameter terms include the AD-PGS, age, and sleep disturbances. Bolded parameters indicate *p <* .05.

**Supplemental Table S13** Parameter Estimates and 95% confidence intervals for ACE Models: AD-PGS (excluding SNPs within the *APOE* region), Age, and Sleep Disturbances as moderators

| Cognitive Task | Est. | 95% CI | Est. | 95% CI | Est. | | 95% CI | Est. | 95% CI |
| --- | --- | --- | --- | --- | --- | --- | --- | --- | --- |
| ***Word List*** | A_p_ | | A_L_ | | C_0_ | | | E_0_ | |
|  | -0.003 | [-0.03, 0.03] | 0.45 | [-0.51, 0.51] | 0.04 | [-0.43, 0.43] | | **0.77** | **[0.74, 0.80]** |
| AD-PGS |  |  |  |  | B_Ap,C_ | | | B_Ap,E_ | |
|  | -- |  | -- |  | 0.08 | | [-0.15, 0.15] | 0.005 | [-0.02, 0.04] |
| Age | B_Age,Ap_ | | B_Age,AL_ | | BA_ge,C_ | | | BA_ge,E_ | |
|  | 0.02 | [-0.02, 0.05] | 0.06 | [-0.11, 0.21] | 0.15 | | [-0.24, 0.24] | -0.01 | [-0.04, 0.03] |
| Sleep Dist. | B_SD,Ap_ | | B_SD,AL_ | | B_SD,C_ | | | B_SD,E_ | |
|  | -0.005 | [-0.03, 0.02] | 0.05 | [-0.20, 0.20] | 0.01 | | [-0.15, 0.15] | -0.03 | [-0.07, 0.01] |
| ***Animal Naming*** | A_p_ | | A_L_ | | C_0_ | | | E_0_ | |
|  | -0.01 | [-0.05, 0.03] | **0.58** | **[0.45, 0.63]** | 0.09 | | [-0.34, 0.34] | **0.72** | **[0.69, 0.76]** |
| AD-PGS |  |  |  |  | B_Ap,C_ | | | B_Ap,E_ | |
|  | -- |  | -- |  | 0.10 | | [-0.20, 0.20] | 0.01 | [-0.02, 0.04] |
| Age | B_Age,Ap_ | | B_Age,AL_ | | BA_ge,C_ | | | BA_ge,E_ | |
|  | -0.004 | [-0.04, 0.04] | 0.01 | [-0.05, 0.07] | -0.01 | | [-0.13, 0.10] | **-0.04** | **[-0.07, -0.003]** |
| Sleep Dist. | B_SD,Ap_ | | B_SD,AL_ | | B_SD,C_ | | | B_SD,E_ | |
|  | 0.03 | [-0.01, 0.07] | -0.02 | [-0.11, 0.06] | 0.12 | | [-0.20, 0.20] | 0.04 | [-0.01, 0.08] |
| ***Digit Forward*** | A_p_ | | A_L_ | | C_0_ | | | E_0_ | |
|  | -0.04 | [-0.07, 0.0003] | **0.60** | **[0.45, 0.69]** | 0.23 | | [-0.38, 0.43] | **0.71** | **[0.67, 0.74]** |
| AD-PGS |  |  |  |  | B_Ap,C_ | | | B_Ap,E_ | |
|  | -- |  | -- |  | 0.01 | | [-0.08, 0.22] | **-0.04** | **[-0.07, -0.01]** |
| Age | B_Age,Ap_ | | B_Age,AL_ | | BA_ge,C_ | | | BA_ge,E_ | |
|  | -0.01 | [-0.05, 0.02] | -0.02 | [-0.08, 0.04] | 0.01 | | [-0.09, 0.19] | -0.01 | [-0.04, 0.02] |
| Sleep Dist. | B_SD,Ap_ | | B_SD,AL_ | | B_SD,C_ | | | B_SD,E_ | |
|  | -0.03 | [-0.06, 0.004] | 0.11 | [-0.10, 0.20] | **-0.14** | | **[-0.24, -0.06]** | -0.03 | [-0.06, 0.03] |
|  |  | |  | |  | | |  | |
| ***Digit Backward*** | A_p_ | | A_L_ | | C_0_ | | | E_0_ | |
|  | -0.01 | [-0.05, 0.02] | **0.60** | **[0.50, 0.65]** | 0.06 | | [-0.18, 0.31] | **0.71** | **[0.68, 0.75]** |
| AD-PGS |  |  |  |  | B_Ap,C_ | | | B_Ap,E_ | |
|  | -- |  | -- |  | 0.07 | | [-0.02, 0.16] | -0.01 | [-0.04, 0.01] |
| Age | B_Age,Ap_ | | B_Age,AL_ | | B_Age,C_ | | | BA_ge,E_ | |
|  | -0.01 | [-0.04, 0.01] | -0.06 | [-0.11, 0.01] | -0.03 | | [-0.12, 0.07] | -0.02 | [-0.05, 0.01] |
| Sleep Dist. | B_SD,Ap_ | | B_SD,AL_ | | B_SD,C_ | | | B_SD,E_ |  |
|  | 0.02 | [-0.02, 0.06] | -0.004 | [-0.14, 0.12] | **0.25** | | **[0.18, 0.31]** | -0.02 | [-0.06, 0.02] |
| ***Synonyms*** | A_p_ | | A_L_ | | C_0_ | | | E_0_ | |
|  | -0.03 | [-0.08, 0.02] | **0.54** | **[0.32, 0.67]** | **0.40** | | **[0.09, 0.58]** | **0.64** | **[0.60, 0.69]** |
| AD-PGS |  |  |  |  | B_Ap,C_ | | | B_Ap,E_ | |
|  | -- |  | -- |  | 0.04 | | [-0.05, 0.18] | -0.02 | [-0.06, 0.02] |
| Age | B_Age,Ap_ | | B_Age,AL_ | | B_Age,C_ | | | BA_ge,E_ | |
|  | -0.01 | [-0.07, 0.05] | **0.24** | **[0.08, 0.38]** | **0.23** | | **[0.06, 0.38]** | **0.12** | **[0.07, 0.17]** |
| Sleep Dist. | B_SD,Ap_ | | B_SD,AL_ | | B_SD,C_ | | | B_SD,E_ | |
|  | -0.02 | [-0.07, 0.02] | 0.06 | [-0.16, 0.16] | -0.05 | | [-0.16, 0.17] | 0.02 | [-0.02, 0.07] |
| ***Symbol Digit*** | A_p_ | | A_L_ | | C_0_ | | | E_0_ | |
|  | -0.04 | [-0.08, 0.01] | **0.68** | **[0.56, 0.73]** | 0.10 | | [-0.36, 0.39] | **0.51** | **[0.47, 0.56]** |
| AD-PGS |  |  |  |  | B_Ap,C_ | | | B_Ap,E_ | |
|  | -- |  | -- |  | 0.07 | | [-0.08, 0.19] | -0.02 | [-0.06, 0.01] |
| Age | B_Age,Ap_ | | B_Age,AL_ | | B_Age,C_ | | | BA_ge,E_ | |
|  | -0.0004 | [-0.04, 0.04] | 0.06 | [-0.01, 0.16] | 0.08 | | [-0.21, 0.21] | 0.02 | [-0.01, 0.06] |
| Sleep Dist. | B_SD,Ap_ | | B_SD,AL_ | | B_SD,C_ | | | B_SD,E_ | |
|  | 0.003 | [-0.03, 0.04] | -0.05 | [-0.14, 0.08] | 0.16 | | [-0.24, 0.24] | 0.02 | [-0.03, 0.06] |

*Note.* A=additive genetic, C=shared environment, E=nonshared environment, PGS-AD-Polygenic Score. Sleep Dist.=Sleep Disturbance. Moderation parameter terms include the AD-PGS, sleep, and age.

**Supplemental Figure 1.** Genetic and environmental variance of Word List, across varying levels of the AD-PGS and varying levels of sleep duration.

|  |  |
| --- | --- |
|  |  |

*Note.* Left panel depicts the AE model and right panel depicts the ACE model. Red Line= latent additive genetic variance, Purple Line= Proportion of genetic variance attributable to the AD-PGS, Green Line= shared environmental variance, Blue Line=unshared environmental variance. Shaded blocks indicate a PGS±2-3 SD’s or Sleep durations ±2-4 from the mean.

**Supplemental Figure 2.** Genetic and environmental variance of Animal Naming, across varying levels of the AD-PGS and varying levels of sleep duration.

|  |  |
| --- | --- |
|  |  |

*Note.* Left panel depicts the AE model and right panel depicts the ACE model. Red Line=latent additive genetic variance, Purple Line= Proportion of genetic variance attributable to the AD-PGS, Green Line= shared environmental variance, Blue Line=unshared environmental variance. Shaded blocks indicate a PGS±2-3 SD’s or Sleep durations ±2-4 from the mean.

**Supplemental Figure 3.** Genetic and environmental variance of Digits Forward, across varying levels of the AD-PGS and varying levels of sleep duration.

|  |  |
| --- | --- |
|  |  |

*Note.* Left panel depicts the AE model and right panel depicts the ACE model. Red Line=latent additive genetic variance, Purple Line= Proportion of genetic variance attributable to the AD-PGS, Green Line= shared environmental variance, Blue Line=unshared environmental variance. Shaded blocks indicate a PGS±2-3 SD’s or Sleep durations ±2-4 from the mean.
